# Advancements and Applications of Injectable Hydrogel Composites in Biomedical Research and Therapy

**DOI:** 10.3390/gels9070533

**Published:** 2023-06-30

**Authors:** Hossein Omidian, Sumana Dey Chowdhury

**Affiliations:** Barry and Judy Silverman College of Pharmacy, Nova Southeastern University, Fort Lauderdale, FL 33328, USA; sd2236@mynsu.nova.edu

**Keywords:** injectable hydrogel nanocomposites, biomedical applications, controlled release, tissue engineering, therapeutic outcomes

## Abstract

Injectable hydrogels have gained popularity for their controlled release, targeted delivery, and enhanced mechanical properties. They hold promise in cardiac regeneration, joint diseases, postoperative analgesia, and ocular disorder treatment. Hydrogels enriched with nano-hydroxyapatite show potential in bone regeneration, addressing challenges of bone defects, osteoporosis, and tumor-associated regeneration. In wound management and cancer therapy, they enable controlled release, accelerated wound closure, and targeted drug delivery. Injectable hydrogels also find applications in ischemic brain injury, tissue regeneration, cardiovascular diseases, and personalized cancer immunotherapy. This manuscript highlights the versatility and potential of injectable hydrogel nanocomposites in biomedical research. Moreover, it includes a perspective section that explores future prospects, emphasizes interdisciplinary collaboration, and underscores the promising future potential of injectable hydrogel nanocomposites in biomedical research and applications.

## 1. Introduction

Injectable hydrogel systems have witnessed noteworthy progress in recent years within the realm of biomedical applications. These biomaterials present a multitude of advantages, including controlled release, targeted delivery, and enhanced mechanical properties [1]. Their potential has been demonstrated in various therapeutic areas, such as cardiac regeneration [1], joint diseases [2], postoperative analgesia [3], and the treatment of ocular disorders [4]. Particularly in the field of tissue engineering, hydrogels play a vital role by promoting crucial aspects such as cell viability, adhesion, differentiation, and host integration [5]. By mimicking the native extracellular matrix in bone and cartilage tissue engineering, hydrogels provide a biocompatible and regenerative environment [5]. Notably, injectable hydrogels enriched with nano-hydroxyapatite have shown promise in bone regeneration [6]. Additionally, collagen-based hydrogels, carboxymethyl-chitosan gels, gelatin, and nano-hydroxyapatite exhibit potential for bone tissue engineering [7,8].

Addressing the clinical challenges associated with bone defects, osteoporosis, and tumor-related bone regeneration represents a significant aspect of injectable hydrogel systems. These biomaterials enhance bone formation and repair by promoting osteogenic differentiation, angiogenesis, and controlled release of bioactive molecules [9,10,11,12,13,14,15,16,17,18,19,20,21,22,23,24,25,26,27]. Various composite biomaterials and injectable hydrogels, such as the RADA16 peptide hydrogel with calcium sulfate/nano-hydroxyapatite cement and the GelMA-HAMA/nHAP composite hydrogel, have demonstrated efficacy in bone healing and regeneration [9,11]. Furthermore, injectable hydrogels incorporating bio-responsive drug-loaded nanoparticles exhibit dual functions and hold promise for targeted delivery, tumor suppression, and bone regeneration [17,18].

Injectable hydrogel systems also find application in wound management and therapy, as they facilitate controlled release of bioactive substances, accelerated wound closure, and tissue regeneration. The incorporation of nanoparticles further enhances their antibacterial activity and bioactivity, advancing the field of wound care [28,29].

In cancer therapy, injectable hydrogel systems offer promising solutions for targeted drug delivery, chemo-photothermal therapy, and combination therapies. These hydrogels’ versatility, controlled-release capabilities, and biocompatibility render them ideal platforms for enhancing therapeutic outcomes [30,31,32,33,34,35,36,37,38,39]. By delivering therapeutic agents and enabling targeted treatments, injectable hydrogels have the potential to significantly advance cancer therapy and improve patient outcomes [40,41].

In addition to their aforementioned applications, injectable hydrogel systems hold great potential in several other areas of biomedical research. These include ischemic brain injury, tissue regeneration, cardiovascular diseases, and personalized cancer immunotherapy [42,43,44,45]. Notably, in the treatment of ischemic brain injury, injectable hydrogels have demonstrated the ability to facilitate neuronal proliferation, angiogenesis, and tissue regeneration, offering promising therapeutic prospects [42]. Similarly, in the realm of cardiovascular diseases, hydrogels combined with stem cells, nanoparticles, or genetic material have shown promise in improving cardiac function and promoting tissue regeneration [44]. Moreover, the integration of nanotechnology and biomaterials in injectable hydrogels has enabled the development of personalized cancer immunotherapy approaches, allowing for targeted and effective treatment modalities [46,47,48,49,50].

Recent studies have also focused on the development of injectable hydrogels for cartilage regeneration [51,52,53]. These hydrogels have shown the capacity to promote cell migration, chondrogenesis, and the expression of cartilage-specific genes, presenting potential solutions for cartilage tissue engineering. Furthermore, functionalization strategies such as kartogenin (KGN)-conjugated nanoparticles and chondroitin sulfate nanoparticles have been employed to enhance the regenerative capabilities of these hydrogels [51,52]. Additionally, the incorporation of nanocrystalline hydroxyapatite has been found to support cell viability and differentiation, further advancing the field of cartilage regeneration [53]. These recent advancements hold promise for improved therapies and treatments in various cartilage-related conditions.

This manuscript provides an overview of the recent advancements and applications of injectable hydrogel nanocomposites in various biomedical fields. It explores their crucial role in drug delivery, tissue engineering, bone regeneration, wound management, and cancer therapy, highlighting the significant contributions these biomaterials make to the advancement of biomedical research and clinical practice.

## 2. Drug Delivery

Over the past few years, hydrogel-based drug delivery systems have made significant strides in providing targeted and controlled release of drugs, thereby holding great potential for enhancing therapeutic outcomes in diverse medical conditions [1]. Hydrogels, with their wide range of properties and synthesis routes, play a crucial role in the realm of controlled drug delivery, rendering them suitable carriers in the field of medicine [54]. For instance, injectable nano-enabled thermogels have demonstrated promise in achieving controlled release of anti-angiogenic peptides for the treatment of ocular disorders, offering extended-release capabilities and biocompatibility [4].

Cardiac regeneration following myocardial infarction (MI) poses challenges due to limited regenerative capacity and scar formation [1]. Tissue engineering approaches, encompassing nano-carriers, controlled release matrices, injectable hydrogels, and cardiac patches, have shown promise in addressing this issue [1]. Similarly, improved drug delivery strategies are required for joint diseases such as osteoarthritis and rheumatoid arthritis. Advanced drug delivery systems, including nano- and microcarriers, have been developed to enhance efficacy and minimize side effects in these conditions [2]. Specific nano-drug delivery systems have been devised for postoperative analgesia, resulting in improved pain relief and better postoperative outcomes [3]. Innovative approaches, such as maleimide-functionalized polyethylene glycol hydrogels loaded with nanoparticles, exhibit potential in preventing cartilage degradation and inhibiting osteophyte formation in the treatment of post-traumatic osteoarthritis [55].

Researchers have explored various drug delivery systems, yielding promising results. For instance, nano hybrid silk hydrogel systems have been developed for localized and targeted delivery of anticancer drugs, demonstrating slow and sustained release as well as active targeting of cancer cells [40]. pH- and temperature-responsive hydrogels incorporating chondroitin sulfate nanogels have exhibited selective binding to lung carcinoma cells and effective inhibition of cancer cell growth [41]. Injectable hydrogel systems equipped with tumor-targeting nano-micelles and glutathione-responsive drug release have demonstrated significant tumor growth inhibition and enhanced antitumor efficacy [56]. These examples underscore the potential of hydrogel-based drug delivery systems in the realm of cancer treatment.

Moreover, hydrogel systems have shown promise in the domain of intravaginal drug delivery for gynecological drugs, contributing to improved drug solubility and distribution [57]. Intravesical liposome-in-gel systems have been developed to enhance drug retention in the bladder while reducing systemic levels, presenting a promising approach for intravesical applications [58]. Similarly, liposome-in-gel-paclitaxel systems have been investigated for regional delivery in chemoradiotherapy, resulting in increased cytotoxicity and reduction in tumor volume [59].

In recent studies, researchers have delved into the exploration of injectable thermosensitive photothermal-network hydrogels capable of near-infrared (NIR)-triggered drug delivery and synergistic photothermal-chemotherapy, showcasing remarkable efficacy in tumor eradication [60]. Additionally, smart and biomimetic 3D- and 4D-printed composite hydrogels have been investigated for their potential in tissue engineering and controlled drug release, offering versatile applications in the field [61]. Ultrasoft polymeric DNA networks (pDNets) with variable crystallinities have been developed as a means of controlled release of anticancer drugs, enabling efficient localized drug delivery and demonstrating significant antitumor efficacy [62]. Moreover, injectable thermoresponsive hydrogels based on graft copolymers have been synthesized to facilitate controlled drug delivery and promote bone cell growth, thus holding promise for applications in regenerative medicine [63]. Furthermore, injectable and degradable polysaccharide-based hydrogels embedded with nanoparticles have exhibited potential in drug delivery and bone tissue engineering endeavors [64].

Smart stimuli-responsive injectable gels and hydrogels have emerged as highly promising tools in the realm of drug delivery and tissue engineering [65]. These systems have shown considerable potential in delivering protein therapeutics for chronic and autoimmune diseases, exemplified by their successful administration of insulin for patients with conditions such as type 1 diabetes mellitus [66]. Moreover, pH-sensitive drug release systems utilizing folic-acid-conjugated graphene oxide have demonstrated targeted delivery of doxorubicin (DOX) in breast cancer therapy. These systems exhibit pH-responsive drug release, enhanced cytotoxicity in vitro, and significant reduction in tumor volume in animal studies, thereby showcasing their potential as effective treatment modalities [67].

Injectable hydrogel-based drug delivery systems have also yielded promising results in various medical applications. For instance, hydrogel nanomaterials employed in continuous subcutaneous insulin infusion (CSII) have shown improvements in blood glucose control and reduced therapeutic time in pediatric patients with type 1 diabetes mellitus [68]. Nanocomposite hydrogels have been investigated for their potential in promoting the healing of diabetic ulcers, capitalizing on their high drug loading capacity and stability, thus offering valuable therapeutic avenues [69]. Disease-responsive drug delivery systems have garnered attention for their improved targeting capabilities and controlled release profiles, contributing to advancements in the field of personalized medicine [70].

Researchers have made notable advancements in the development of injectable hydrogels with diverse properties and functionalities. For instance, an injectable, self-healing, and pH-responsive nanocomposite hydrogel has been devised, displaying potential applications in cancer therapy, wound healing, and infection treatment, thereby presenting a versatile platform for various medical interventions [71]. Similarly, an injectable liquid metal nanoflake hydrogel has been designed to enhance postsurgical tumor recurrence suppression, displaying long-term antitumor effects while mitigating systemic toxicity [72]. Furthermore, a bio-inspired fluorescent nano-injectable hydrogel with sequential drug release capabilities has been proposed, offering potential applications in visualization and dual drug delivery, thus opening new avenues for therapeutic interventions [73]. Lastly, an injectable micromotor@hydrogel drug delivery system has been developed for antibacterial therapy, exhibiting antibacterial effects and enhanced activity near bacteria without the need for exogenous hydrogen peroxide, showcasing its potential in combating bacterial infections [74].

Other research studies have concentrated on specific therapeutic domains, aiming to address specific medical needs. To facilitate angiogenic drug delivery, a nano polydopamine (PDA) crosslinked thiol-functionalized hyaluronic acid (HA) hydrogel has been developed, showcasing an injectable nature and sustained drug release capabilities [75]. For the repair of spinal cord injuries (SCI), drug delivery systems have been optimized utilizing sustained-release microspheres loaded with melatonin (Mel) and Laponite hydrogels, enabling stable and prolonged release for neural function restoration [76].

**Figure 1:**
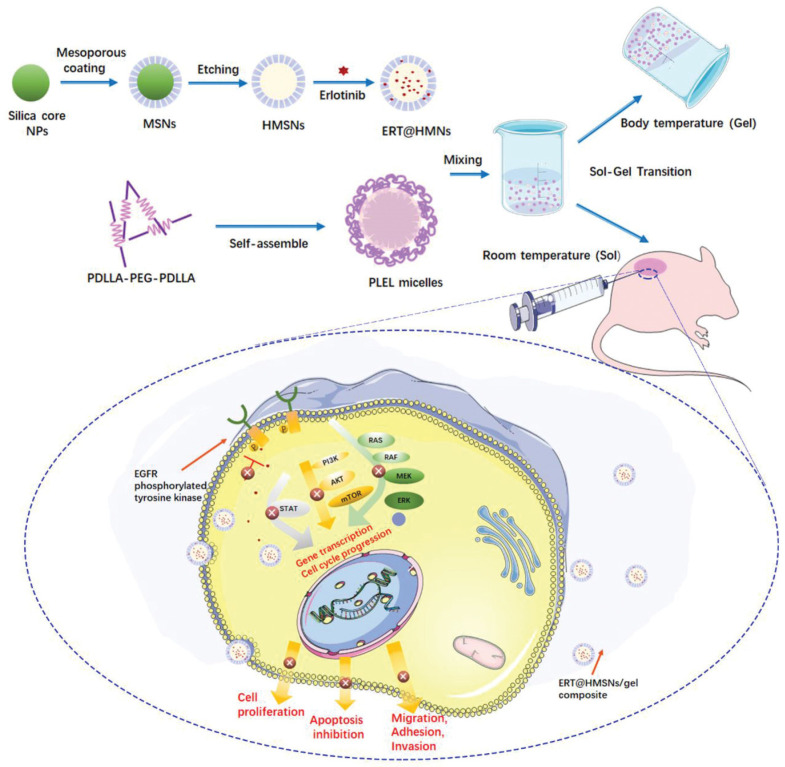
Schematic representation of ERT@HMSNs/gel composite to treat NSCLC. Hollow mesoporous silica nanoparticles (HMSNs) act as a carrier for encapsulating erlotinib, aiming to enhance its therapeutic efficacy and mitigate drug-related toxicity. To endure homogeneity and stability of the injectable matrix, PLEL added to the ERT@HMSNs solution. The evaluation included assessing transition from sol to gel phase and the sustained release of the drug. Adopted with permission [77].

**Figure 2:**
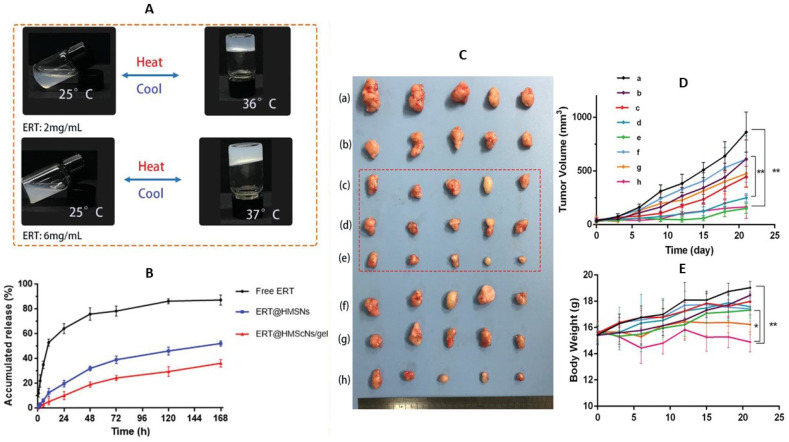
(**A**) Reversible sol–gel phase transition of ERT@HMSNs/gel composite; (**B**) In vitro drug release profile; (**C**) In vivo antitumor efficiency of different ERT formulations and Terceva on NSCLC xenograft models. (a) NS; (b) ERT@HMSNs; (c–e) different concentrations of ERT@HMSNs/gel (25, 50, and 100 mg/kg); (f–h) different concentrations of marketed drug Tarceva (25, 50, and 100 mg/kg). (**D**) The tumor growth curves of each group. (**E**) Body weight changes of mice as a function of time in each group. All quantitative data are given as mean ± SD (n = 5). “*” mean *p* < 0.05 and “**”mean *p*< 0.01. Adopted with permission [77].

In the localized treatment of non-small cell lung cancer (NSCLC), an injectable thermosensitive hydrogel composed of the amphiphilic triblock copolymer poly(d, l-lactide)-poly(ethylene glycol)-poly(d, l-lactide) loaded with erlotinib-loaded hollow mesoporous silica nanoparticles (ERT@HMSNs/gel) has exhibited promising outcomes (Figure 1) [77]. This investigation involved determining the sol-to-gel transition temperature, which corresponds to the body temperature, as well as evaluating in vitro drug release from HMSNs with or without the gel. Moreover, an in vivo comparative study of different ERT formulations and the commercially available drug Tarceva was conducted using NSCLC xenograft models (Figure 2). The findings revealed that the ERT-loaded HMSNs/gel system displays substantial potential as an in-situ treatment approach for NSCLC, offering prolonged drug retention along with efficacy and safety. Similarly, an injectable silk fibroin nanofiber hydrogel for vancomycin delivery has demonstrated exceptional antibacterial properties and biocompatibility [78].

Polysaccharide-based nanoscale drug delivery systems have garnered significant attention within the field of tissue engineering due to their potential applications. These systems leverage the unique properties of polysaccharides to mimic the extracellular matrix and modulate cellular functions [79]. A recent perspective article discussed the clinical relevance and future prospects of an injectable hydrogel and nanoparticle system designed for microRNA (miR) delivery to the heart, emphasizing its potential in cardiac therapy [80]. In another study, researchers explored a composite hydrogel incorporating P24 peptide-loaded microspheres and nano-hydroxyapatite, demonstrating promising outcomes for bone regeneration in tissue engineering [81]. Similarly, a thermosensitive injectable hydrogel based on PLGA-PEG-PLGA copolymer, integrated with hydroxyapatite particles, showcased controlled release of calcium cations, thus exhibiting potential for calcium delivery in bone regeneration [82].

Silica microparticles encapsulating triptorelin acetate formed an injectable depot with sustained release characteristics, exhibiting pharmacodynamic effects comparable to those of commercially available products [83]. The controlled release of bone morphogenetic protein-2 (BMP-2) from a three-dimensional tissue-engineered nano-scaffold resulted in significant ectopic bone formation, further underscoring its potential for tissue regeneration [84]. A multifunctional sustainable delivery system for calcitriol, employing a thermosensitive hydrogel, nano-hydroxyapatite, and calcitriol-loaded micelles, stimulated osteogenesis and bone regeneration both in vitro and in vivo, displaying low cytotoxicity and an appropriate degradation rate [85]. Additionally, a thermosensitive micellar hydrogel composed of PELT triblock copolymer exhibited potential as an injectable nanomedicine reservoir and platform for co-delivery of therapeutic agents [86]. Lastly, a macroscale thermosensitive micellar-hydrogel depot demonstrated sustained drug release, stable immobilization of radioisotopes, and enhanced antitumor effects, thus offering a promising approach for combined chemoradiotherapy [87].

In the realm of drug delivery systems, innovative approaches have been explored, such as a cell-penetrable nano-polyplex hydrogel system that enables localized siRNA delivery, thereby facilitating long-term and site-specific gene silencing through a single injection [88]. Furthermore, a moldable and biodegradable colloidal nano-network was developed for the protection and localized delivery of antimicrobial peptides, highlighting its potential in preserving bioactivity and effectively eliminating bacteria [89].

Efforts to promote neuroregeneration following traumatic spinal cord injury have been a major focus in therapeutic delivery strategies. Injectable hydrogel-based systems and scaffold architectures have demonstrated promising results in facilitating neuroregeneration [90]. Graphene oxide (GO) has been extensively investigated as a nanofiller in self-assembling peptide hydrogels for intervertebral disc repair, exhibiting mechanical properties akin to those of the nucleus pulposus while supporting cell viability [91]. Alginate nanohydrogels loaded with bone morphogenetic protein-2 (BMP-2) have been proposed as a controlled release system to enhance osteoblastic growth and differentiation [92]. 

In the realm of antioxidant therapy, a hydrogel delivery system incorporating curcumin into oligo-conjugated linoleic acid vesicles (OCLAVs) and a chitosan (CS) hydrogel demonstrated sustained release of curcumin over a span of 96 h, accompanied by enhanced antioxidant activity [93]. Similarly, a study focused on an injectable hydrogel composed of chitosan, collagen, hydroxypropyl-gamma-cyclodextrin, and polyethylene glycol, which showcased a two-step forming process and controlled release of bioactive substances [94]. These findings underscore the potential of hydrogel systems in antioxidant therapy, offering prolonged release and improved therapeutic effects.

Advances in the field of bone regeneration have led to the synthesis of poly(phosphazene) hydrogels with specific release rates, demonstrating their efficacy in promoting bone regeneration [95]. Additionally, a noteworthy development is the injectable hydrogel depot system that employs sustained release of exendin 4 (Ex-4) for the treatment of type 2 diabetes mellitus [96]. These studies exemplify the versatility of hydrogel-based systems in controlled release and their potential in bone regeneration and diabetes treatment.

Enhancing the mechanical properties of injectable hydrogels has been a significant focus in the context of bone regeneration. The incorporation of nano-hydroxyapatite or strontium hydroxyapatite, along with dopamine modification, has been shown to augment the mechanical properties of alginate-based hydrogels [97]. Furthermore, the injectable bone regeneration composite (IBRC), which facilitates controlled release of recombinant human bone morphogenetic protein-2 (rhBMP-2), has demonstrated potential for clinical applications in bone defect repair [98]. These advancements underscore the promise of injectable hydrogel systems in the realms of bone regeneration and tissue engineering.

In the pursuit of improved drug delivery, nanoparticle-based systems have been explored for the enhanced delivery of nitric oxide (NO) in cancer treatment [99]. Injectable nano-apatite scaffolds, capable of delivering osteogenic cells and growth factors, have exhibited promise in promoting bone regeneration [100]. Moreover, injectable quadruple-functional hydrogels have demonstrated enhanced tumor targeting and significant reduction in tumor volume through sustained targeting and combined therapy approaches [101]. Additionally, the development of a multiple magnetic hyperthermia (MHT)-mediated release system utilizing an injectable, thermosensitive polymeric hydrogel has proven effective in combination cancer therapy [102]. These studies shed light on the potential of injectable hydrogel systems in cancer therapy, bone regeneration, and controlled release of therapeutic agents.

Overall, the ongoing advancements in hydrogel-based systems continue to drive innovation in the field of drug delivery, providing versatile platforms for controlled release, targeted delivery, and improved therapeutic outcomes in various biomedical applications. These advancements pave the way for enhanced patient care and treatment outcomes. Table 1 provides an overview of the injectable hydrogel materials employed primarily in drug delivery systems.

Injectable hydrogel systems have emerged as a highly promising platform for drug delivery, demonstrating notable advantages in achieving localized and sustained release, thereby facilitating targeted therapy. This feat is accomplished through the implementation of various strategies, such as the utilization of functional ions, nanomaterials, and responsive systems, which collectively contribute to the achievement of controlled release profiles. Furthermore, the integration of hybrid formulations, nanogels, liposome-in-gel systems, and thermo-responsive hydrogels effectively ensures prolonged drug release. Exciting prospects are also observed in combination therapies, where the amalgamation of drug delivery systems with complementary agents exhibits synergistic effects, promising enhanced therapeutic outcomes. In the specific context of bone tissue regeneration, hydrogels play a pivotal role by incorporating bioactive components such as peptides, calcium cations, micelles, and growth factors, thereby promoting osteogenesis and bone formation. Moreover, the tunability of mechanical properties in hydrogels facilitates cell proliferation and provides an adaptable environment for tissue engineering applications.

## 3. Tissue Engineering

In recent years, significant advancements have been made in the field of tissue engineering, particularly in the development of functional hydrogel-based constructs for tissue regeneration. Researchers have leveraged advancements in nano-based 3D and 4D scaffolds, stem cells, and biomaterial innovations to achieve promising results in various applications, including bone and cartilage tissue engineering [5]. Notably, electrospun nanofibrous scaffolds and hydrogel scaffolds that emulate the native extracellular matrix have substantially improved cell viability, adhesion, differentiation, and host integration in these areas [5]. Furthermore, aligned conductive core-shell biomimetic scaffolds have emerged as potential tools for peripheral nerve tissue regeneration [106].

In the realm of bone regeneration, injectable thermosensitive hydrogels enriched with nano-hydroxyapatite have demonstrated potential as biocompatible alternatives [6]. Researchers have tackled challenges associated with natural hydrogels by developing efficient encapsulation systems employing bivalent cobalt-doped nano-hydroxyapatite and gum tragacanth for bone tissue engineering [107]. Additionally, injectable collagen-based hydrogels with controlled mechanical properties have shown promise in bone regeneration applications [7].

The biocompatibility and immune response of injectable collagen/nano-hydroxyapatite (Col/nHA) hydrogels have been investigated for hard tissue engineering [108]. Injectable gels composed of carboxymethyl-chitosan, gelatin, and nano-hydroxyapatite have also demonstrated potential in bone tissue engineering [8]. Similarly, hydrogel constructs incorporating chondroitin sulfate nanoparticles and nano-hydroxyapatite have exhibited superior properties for osteochondral regeneration [109]. Hydrogels have also been recognized as promising vehicles for cardiac tissue regeneration, offering minimally invasive administration and effective delivery of therapeutic agents [110].

Polymeric scaffolds, including hydrogels with nano-additives, have emerged as solutions to address limitations in bone/cartilage and neural tissue engineering [111]. For instance, the addition of zirconium oxide nanoparticles to alginate-gelatin hydrogels has enhanced their mechanical properties and regulation of biodegradation, rendering them suitable for cartilage tissue engineering [112]. Injectable alginate-O-carboxymethyl chitosan/nano fibrin composite hydrogels have been developed for adipose tissue engineering, supporting stem cell proliferation and differentiation [113]. Carrageenan nanocomposite hydrogels incorporating whitlockite nanoparticles and an angiogenic drug have also shown promise for bone tissue engineering [114].

Poly(ethylene glycol)-poly(epsilon-caprolactone)-poly(ethylene glycol) nanocomposites with nano-hydroxyapatite demonstrate thermoresponsivity and favorable gelation properties, making them viable options for orthopedic tissue engineering [115]. Improving the mechanical strength and bioactivity of chitosan/collagen hydrogels through the integration of functionalized single-wall carbon nanotubes holds promise for bone regeneration [116]. Composite hydrogels incorporating nano-hydroxyapatite, glycol chitosan, and hyaluronic acid present porous structures and cytocompatibility, making them attractive for bone tissue engineering [117]. Similarly, a composite hydrogel combining laponite nanoparticles and silated hydroxypropylmethyl cellulose exhibits improved mechanical properties and cytocompatibility for cartilage tissue engineering [118]. Additionally, a biohydrogel incorporating nano SIM@ZIF-8 demonstrates osteogenic differentiation and lipid-lowering abilities, offering potential for bone repair [119].

**Figure 3:**
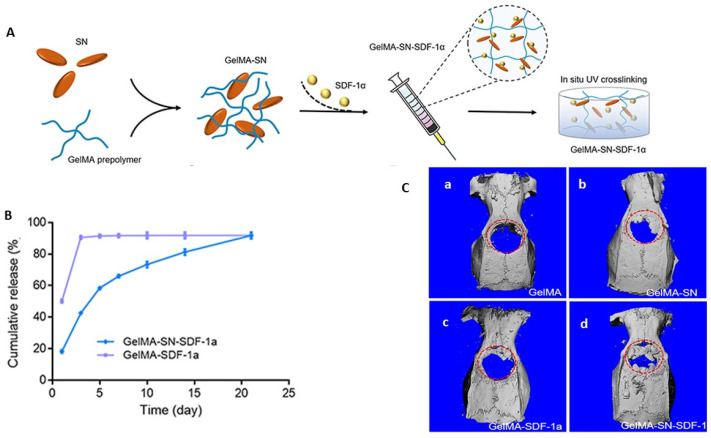
(**A**) Schematic presentation of GelMA-SN-SDF-1α hydrogel fabrication; (**B**) In vitro study of release profile of SDF-1α from GelMA-SDF-1α and GelMA-SN- SDF-1α and (**C**) Micro-CT scanning results of the bone healing of calvaria defects rats treated with GelMA, GelMA-SN, GelMA-SDF-1α, and GelMA-SN-SDF-1α hydrogel for 6 weeks. Adopted with permission [120].

In the field of tissue engineering, researchers have developed innovative approaches for fabricating injectable hydrogels with diverse applications [120,121,122,123,124,125,126,127,128]. One noteworthy example is an osteogenic hydrogel composed of gelatin-methacryloyl pre-polymer (GelMA) and nano silicate (SN) (Figure 3A). This hydrogel has shown promising results in in vitro studies on SDF-1α release and in vivo studies on a rat calvaria defect model (Figure 3B,C) [120]. The GelMA-SN-SDF-1α hydrogel exhibits injectability, controlled release of SDF-1α, and the ability to stimulate mesenchymal stem cell migration and expression of osteogenic-related biomarkers.

The field of regenerative medicine has witnessed notable advancements in nanoengineered biomimetic hydrogels, particularly in the realm of 3D printing. These hydrogels exhibit improved mechanical properties and create an interactive environment that facilitates favorable outcomes in tissue regeneration [121]. Furthermore, the utilization of mineralized heparin-gelatin nanoparticles has shown promise in bone tissue engineering, serving as versatile fillers or multifunctional devices for nanotherapeutic approaches [122].

In the context of hair follicle tissue engineering, the application of GelMA/chitosan-microcarriers loaded with platelet-rich plasma and dermal papilla cells has yielded encouraging results, promoting hair follicle growth and vascularization to enable hair regeneration [123]. Injectable hydrogel composites based on polysaccharides and incorporating nano-hydroxyapatite have been developed as scaffolds for bone tissue engineering, exhibiting remarkable efficacy in bone repair [124]. Similarly, bioactive glass nanoparticle-reinforced injectable hydrogels composed of PEG and pNVC copolymers have demonstrated improved properties and enhanced osteogenesis, positioning them as suitable grafting materials for orthopedic reconstructive surgeries [125]. Additionally, an injectable hydrogel composed of collagen fibrils and a glycol-chitosan matrix has been tailored to address the unique challenges of mechanically strained tissues in soft tissue engineering [126].

Researchers have underscored the significance of integrating stem cells, nanotechnology, and biomaterial innovations in the development of functional hydrogel-based constructs for tissue regeneration [127]. Notably, visible-light-mediated nano-biomineralization has emerged as a rapid fabrication method for customizable biomineralized tough hydrogels, exhibiting enhanced mechanical and biological properties. These hydrogels hold great promise for applications in skin repair and bone regeneration [128].

The collective efforts in hydrogel development offer significant potential for advancing the field of tissue engineering and regenerative medicine. For a comprehensive overview of the hydrogel materials utilized in tissue engineering, please refer to Table 2.

These studies underscore the versatility and potential of hydrogels in tissue engineering and regenerative medicine. By customizing the composition, structure, and properties of hydrogels, researchers can design materials that promote cell alignment, controlled release of bioactive substances, integration of nanoparticles, and interpenetrating network structures, all of which contribute to their suitability for various tissue engineering applications.

## 4. Bone Repair

In recent years, considerable research endeavors have been devoted to the advancement of biomaterials tailored specifically for bone regeneration purposes. The primary objective of these biomaterials is to promote bone formation and address various bone defects, thereby offering potential solutions for a wide range of clinical applications. Among the emerging avenues in this field, composite biomaterials and injectable hydrogels have garnered significant attention.

Composite biomaterials, such as the combination of RADA16 peptide hydrogel with porous calcium sulfate/nano-hydroxyapatite (CaSO_4_/HA) cement, have emerged as a promising approach. Notably, this particular composite has demonstrated improved osteogenic differentiation and enhanced bone formation in femoral condyle defects [9]. Another notable study developed an injectable hydrogel for craniofacial bone regeneration, incorporating bioglass or whitlockite nanoparticles with FGF-18 into a chitin-PLGA hydrogel. This hydrogel exhibited sustained release of FGF-18, resulting in near-complete bone regeneration in craniofacial bone defects [10].

The development of injectable hydrogels has also shown promise as a viable strategy for bone regeneration. For instance, a GelMA-HAMA/nHAP composite hydrogel encapsulating human-urine-derived stem cell exosomes (USCEXOs) exhibited controlled-release properties, fostering osteogenesis and angiogenesis in vitro, while significantly enhancing cranial bone defect repair in a rat model [11]. Additionally, injectable bone regeneration composites composed of nano-hydroxyapatite/collagen (nHAC) particles within an alginate hydrogel carrier demonstrated controllable degradation, biocompatibility, and great potential for bone repair and tissue engineering [12].

**Figure 4:**
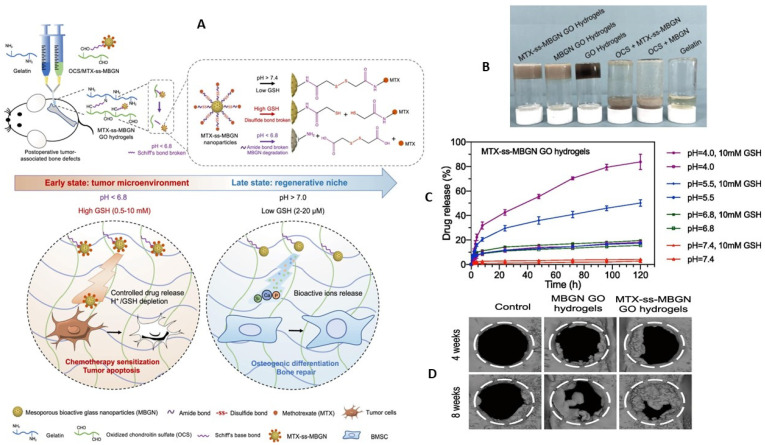
(**A**) Schematic representation of the synthesized injectable TME-modulated MTX-ss-MBGN GO hydrogels showing residual tumor apoptosis, restoring chemotherapy sensitivity and promoting bone regeneration for postoperative tumor-associated bone defect closed-loop management; (**B**) Observation study of hydrogels; (**C**) In vitro drug release study to confirm controlled release capability of MTX-ss-MBGN GO hydrogels under tumor-mimicking environment; (**D**) Micro-CT image of calvarial defect repair at 4 or 8 weeks under different treatments on healthy SD rats. Adopted with permission [17].

Noteworthy advancements in the realm of biomaterials for bone regeneration also encompass injectable hydrogels with dual functionality. One particular study focused on tumor microenvironment-modulated hydrogels (TME), which incorporated bio-responsive drug-loaded mesoporous bioactive glass nanoparticles (MTX-ss-MBGN), gelatin, and oxidized chondroitin sulfate (OCS) for the treatment of tumor-associated bone defects (Figure 4A). The injected Gel/OCS solution, along with MTX-ss-MBGN, rapidly formed a TME-modulated hydrogel, facilitating sustained drug-responsive release with targeted delivery to tumor cells through the depletion of protons and glutathione (GSH). Figure 4B,C depict the hydrogel formation process and the dual responsiveness, highlighting the controlled release capability of MTX-ss-MBGN GO hydrogels. Moreover, this hydrogel facilitated bone regeneration by transitioning into regenerative scaffolds (Figure 4D), effectively preventing tumor recurrence [17]. Similarly, an injectable hydrogel utilizing cisplatin and polydopamine-decorated nano-hydroxyapatite demonstrated tumor ablation, suppression of tumor growth, and improved adhesion and proliferation of bone mesenchymal stem cells [18].

Recent scientific investigations have placed significant emphasis on the advancement of injectable gel systems and hydrogel composites designed for bone regeneration, with the aim of addressing a wide range of clinical applications. Noteworthy examples include the utilization of chitin-CaSO_4_-nano-fibrin gel and a double cross-linked hydrogel consisting of gelatin, alginate dialdehyde, calcium ions, and nano-sized hydroxyapatite. These composite hydrogels have demonstrated improved angiogenesis, osteogenesis, and efficacy in bone defect repair [21,23].

Furthermore, the development of injectable hydrogel systems specifically tailored for bone regeneration has garnered considerable attention in research endeavors. One such system involves the incorporation of controlled-release microspheres of bone morphogenetic protein 2 (BMP-2) and 17 beta-estradiol within a composite hydrogel, showcasing uniform discharge and promising outcomes in tissue regeneration [24]. In another study, chitin nano-whiskers (CNWs) were integrated into a chitosan/beta-glycerophosphate disodium salt (CS/GP) hydrogel, resulting in enhanced mechanical properties, improved biocompatibility, and a controlled drug release rate [25].

Moreover, an injectable nano-composite hydrogel for bone regeneration was developed by facilitating the in-situ growth of calcium phosphate (CaP) nanoparticles. This approach exhibited superior mechanical properties, enhanced cell adhesion, osteodifferentiation, and noteworthy advancements in bone formation in vivo [26]. Similarly, an injectable bone regeneration composite (IBRC) employing a calcium alginate hydrogel matrix carrying nano-hydroxyapatite/collagen particles demonstrated structural homogeneity, histocompatibility, and bone healing capabilities [27].

The potential of injectable hydrogels in promoting bone formation, enhancing bone architecture, and addressing bone-related conditions has been substantiated by various studies. For instance, a rabbit-based investigation utilized a biomimetic/osteoinductive injectable hydrogel comprising hyaluronan and nano-hydroxyapatite crystals, leading to substantial improvements in bone density and architecture [130]. Another study compared the osteogenic potential of two injectable hydrogels, namely, demineralized dentin matrix (DDM) hydrogel and nano-hydroxyapatite (n-HA), with the DDM hydrogel demonstrating promising outcomes in promoting collagen-I gene expression and alkaline phosphatase activity [131]. Additionally, an injectable hydrogel based on gellan gum (GG) loaded with chlorhexidine (CHX) and nano-hydroxyapatite (nHA) exhibited a three-dimensional polymeric network, remarkable biocompatibility, and notable osteogenic properties, effectively inhibiting bacterial growth and presenting a potential treatment option for infectious bone defects [132]. A comprehensive summary of the hydrogel materials employed in tissue engineering can be found in Table 3.

These studies underscore the potential of hydrogels in bone tissue engineering and regeneration. By leveraging controlled-release mechanisms, ensuring biocompatibility and tissue integration, enhancing mechanical properties, and addressing specific clinical challenges such as bone defects and infections, hydrogels hold promise as materials for promoting bone healing, regeneration, and broader tissue engineering applications.

## 5. Wound Healing

Considerable advancements have been made in the realm of hydrogel-based materials for wound healing and therapy. These versatile hydrogels have garnered significant attention in the context of wound dressings due to their ability to address multiple aspects of wound management. A comprehensive review conducted in this domain highlights the numerous benefits associated with hydrogel dressings, including moisture retention, protective qualities, exudate absorption, as well as anti-inflammatory and antibacterial properties [133]. The review also delves into the feasibility and future trends concerning hydrogel dressing development, encompassing diverse preparation materials, cross-linking methods, and hydrogel types.

In a particular study, researchers explored the incorporation of catechol-modified quaternized chitosan (QCS-C) within a poly(d,l-lactide)-poly(ethylene glycol)-poly(d,l-lactide) (PLEL) hydrogel. This integration resulted in improved tissue adhesion and a reduced gelation temperature. Moreover, the inclusion of nano-scaled bioactive glass (nBG) further augmented angiogenesis and accelerated wound healing [134].

Chitosan-carboxymethyl cellulose (CMC)-based hydrogels loaded with nano-curcumin exhibited controlled release properties, cytocompatibility, and facilitated tissue regeneration, making them well-suited for diabetic wound repair [135].

A noteworthy study in the field involves the development of a black nano-titania thermogel, wherein nanosized black titania nanoparticles were integrated into a chitosan matrix. This composition highlighted simultaneous photothermal and photodynamic therapy effects. Furthermore, it supported normal skin cell functions and promoted the regeneration of skin tissue, thereby facilitating the healing of cutaneous tumor-induced wounds [136].

Regarding antibacterial activity and wound healing, an injectable silver-gelatin-cellulose hydrogel dressing, incorporated with silver nanoparticles, exhibited enhanced wound-healing properties, particularly in the context of infant nursing care [137]. Additionally, a berberine-modified ZnO nano-colloid hydrogel demonstrated exceptional moisturizing capabilities, anti-inflammatory effects, and notable wound healing abilities, positioning it as a promising candidate for diabetic wound healing [138]. Another intriguing hydrogel formulation involved the encapsulation of Ag-decorated polydopamine nanoparticles within a cationic guar gum hydrogel, exhibiting high photothermal conversion efficiency and potent antibacterial properties, thereby presenting a potential solution for wound healing and combatting bacterial infections [139].

Considerable advancements have been achieved in the realm of hydrogel-based materials specifically designed for wound-healing applications. Notably, the incorporation of nanoparticles has emerged as a valuable approach for enhancing the properties of these hydrogels. For instance, an alginate nanocomposite hydrogel incorporating nano-sized calcium fluoride particles has demonstrated enhanced bioactivity and antibacterial properties. This hydrogel formulation effectively promotes fibroblast cell proliferation, facilitates cell migration, and accelerates the process of wound healing [28].

Gellan gum methacrylate and laponite nanocomposite hydrogels have also exhibited notable improvements in mechanical properties and swelling behavior. These hydrogels hold potential as carriers for therapeutic agents, making them well-suited for the treatment of chronic infections in burn wounds [29]. In another study, researchers introduced an injectable nano-composite hydrogel composed of curcumin, N,O-carboxymethyl chitosan, and oxidized alginate. This hydrogel formulation demonstrates controlled release behavior and expedites wound healing in vivo, positioning it as a highly promising material for wound dressings [140].

**Figure 5:**
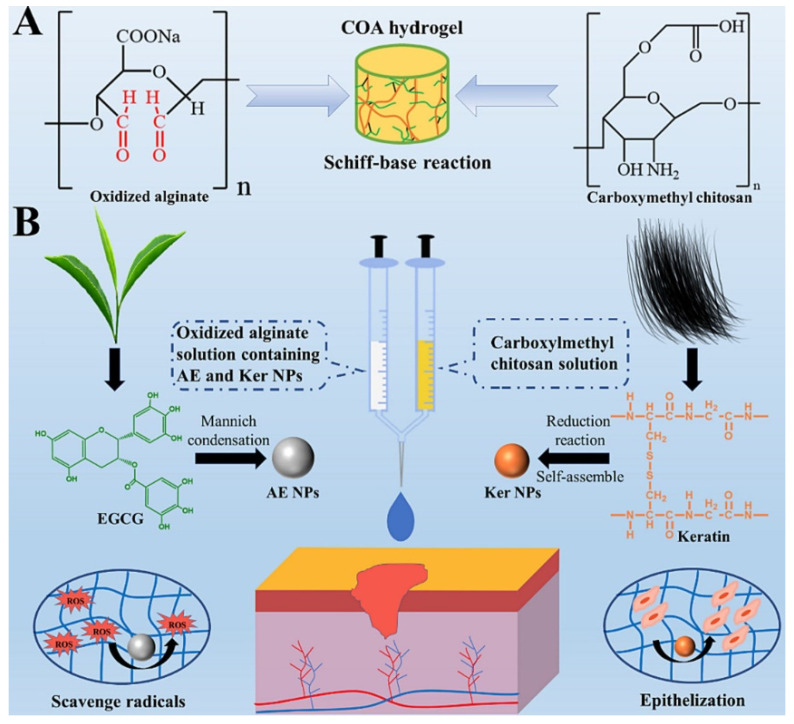
(**A**,**B**) Schematic representation of COA hydrogel fabrication and functionalization for wound repair. Adopted with permission [141].

**Figure 6:**
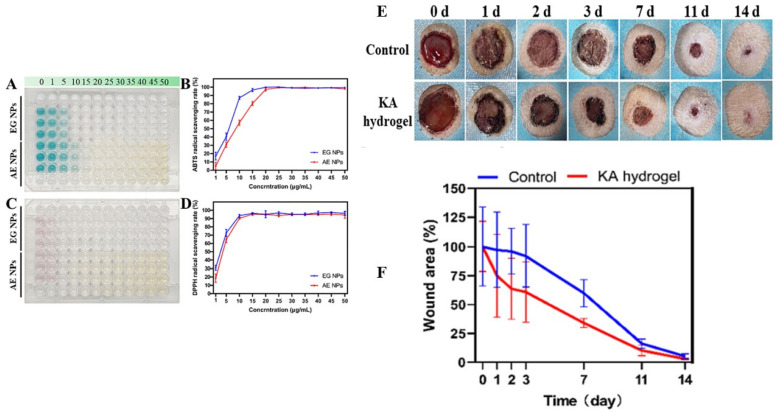
Images and quantitative antioxidant capacity of (**A**,**B**) ABTS and (**C**,**D**) DPPH radical scavenging assay of EG and AE NPs. (**E**) Photographs of the wound area of rats treated with or without KA hydrogel at different times. (**F**) Statistical results of wound area at different times where the wound area for KA hydrogel is 34% decreased than control group at day 7. Adopted with permission [141].

Furthermore, a multifunctionalized injectable hydrogel, known as COA hydrogel, has garnered attention for its exceptional wound-repair capabilities. Comprising oxidized alginate/carboxymethyl chitosan (KA hydrogel) integrated with keratin nanoparticles (Ker NPs) and nanosized-EGCG covered with silver nanoparticles (AE NPs), this hydrogel displays promising outcomes. It promotes epithelization, scavenges radicals, and exhibits significant improvements in wound healing, as substantiated by an increase in epidermis thickness [141]. Please refer to Figure 5 for an illustration of the multifunctionalized injectable hydrogel (COA hydrogel) and Figure 6 for visual evidence of its efficacy in promoting wound healing.

An exemplary instance involves the utilization of an injectable hydrogel system that combines methacryloxylated silk fibroin, metformin-loaded mesoporous silica microspheres, and silver nanoparticles. This nano-dressing exhibits remarkable immunomodulatory effects, effectively inhibits bacterial growth, enhances fibroblast migration, and promotes angiogenesis. Consequently, it emerges as a highly promising solution for the treatment of diabetic wounds [142].

Injectable hydrogel dressings also show great potential for surgical applications. For instance, a hydrogel adhesive possessing rapid adhesion and anti-swelling properties holds promise for achieving prompt hemostasis and wound sealing [143]. Furthermore, a sodium alginate-based injectable hydrogel dressing, cross-linked with gallic acid-functionalized silver nanoparticles, exhibits sustained antimicrobial activity, reduces the inflammatory response, and accelerates wound healing in infected wounds [144].

Numerous novel injectable hydrogel dressings have been developed with outstanding antibacterial properties and enhanced wound-healing capabilities. These include injectable hydrogels incorporating Ag-doped Mo2C-derived polyoxometalate nanoparticles, fusiform-like zinc oxide nanorods, and mce-like Au-CuS heterostructural nanoparticles [145,146,147].

In a separate study, the therapeutic potential of an injectable hydrogel composed of nano-sized suspended formulations of human fibroblast-derived matrix (sFDM) is explored. This hydrogel demonstrates excellent biocompatibility, mechanical stability, and regenerative effects, effectively promoting wound healing, neovessel formation, and reducing necrosis and fibrosis in preclinical models [148].

The development of multifunctional hydrogels incorporating bioactive silver-lignin nanoparticles shows considerable promise for managing chronic wounds. These hydrogels possess commendable antimicrobial, antioxidant, and tissue remodeling properties [149]. Similarly, dual-functional hydrogels composed of guar gum-grafted-polyacrylamidoglycolic acid and silver nanocomposites exhibit self-healing capabilities, injectability, and bacterial inactivation properties, rendering them highly advantageous for wound-healing applications [150].

These innovative hydrogel-based materials present exciting opportunities for wound management and therapy. For further details and a comprehensive overview of various hydrogel materials and their outcomes, please refer to Table 4.

The aforementioned studies serve as significant indicators of the potential that hydrogels hold in the realm of wound healing applications. With their inherent thermo-sensitivity, controlled release capabilities, antibacterial properties, and ability to facilitate various wound-healing processes, hydrogels offer a promising avenue for accelerating the healing of wounds, managing infections, and promoting tissue regeneration. The versatile formulation and properties of hydrogels further contribute to their efficacy in wound care. As research and development in this field progresses, the role of hydrogel-based materials in enhancing wound care and improving patient outcomes is expected to be further enhanced.

## 6. Photothermal

The use of injectable hydrogels has emerged as a promising approach for various modalities of cancer therapy. Specifically, studies have focused on developing injectable hydrogel systems for chemo-photothermal therapy, targeted drug delivery, and combination therapies.

In the realm of chemo-photothermal therapy, innovative hydrogel formulations have been developed to enhance therapeutic outcomes. For instance, a study investigated an in-situ-forming hydrogel incorporating dopamine-reduced graphene oxide (DOPA-rGO) and resveratrol for breast cancer therapy [30]. Another study developed an in-situ-injectable PEG hydrogel system loaded with albumin nanoparticles, demonstrating efficient singlet oxygen generation and hyperthermia, resulting in enhanced cancer cell killing [31]. Furthermore, injectable and biodegradable nano-photothermal DNA hydrogel nanoparticles were engineered to improve tumor cell sensitivity to photothermal and photodynamic treatments [32]. These studies highlight the potential of injectable hydrogels in chemo-photothermal therapy, providing targeted and effective options for cancer treatment.

Moreover, injectable hydrogels have been explored for targeted drug delivery and combination therapies. A self-healing nanocomposite hydrogel carrying graphene oxide (GO) and nano-hydroxyapatite (HAP) exhibited tumor inhibition and photothermal effects, offering a potential treatment for tumors without the side effects of chemotherapy [33]. An intelligent thermo-responsive hydrogel system loaded with berberine hydrochloride (BH) demonstrated enhanced anti-tumor activity when combined with laser irradiation [34].

Recent advancements have expanded the applications of injectable hydrogels in cancer therapy. For example, an injectable nano-composite hydrogel based on hyaluronic acid-chitosan derivatives demonstrated simultaneous photothermal-chemotherapy of cancer with anti-inflammatory capacity, exhibiting favorable tumor inhibition effects [35]. A silk fibroin nanofiber hydrogel system complexed with upconversion nanoparticles and nano-graphene oxide showed excellent biocompatibility and efficient cancer cell ablation through upconversion luminescence imaging and photothermal therapy [36]. Additionally, an injectable and near-infrared (NIR)/pH-responsive nanocomposite hydrogel incorporated gold nanorods, enabling sustained drug release and offering therapeutic potential for chemophotothermal synergistic cancer therapy [37].

Furthermore, the development of bifunctional biomaterials has been explored for bone tumor therapy, combining tumor photothermal therapy with enhanced bone regeneration [38]. The utilization of injectable thermosensitive hydrogels loaded with deferasirox nanoparticles presented a potential strategy for combined chemo-photothermal therapy in melanoma, offering localized drug delivery and photothermal therapy [39]. Please refer to Table 5 for a description of the hydrogel materials used in photothermal therapy.

These studies highlight the potential of hydrogels in cancer therapy by providing a platform for synergistic treatments, controlled drug release, tumor ablation, and proliferation control. By leveraging these capabilities, hydrogels offer promising strategies for improving therapeutic efficacy and reducing adverse effects in cancer treatment.

## 7. Other Biomedical Applications

### 7.1. Angiogenesis

Injectable hydrogels have emerged as versatile biomedical tools with wide-ranging applications, including the treatment of ischemic brain injury, cardiovascular diseases, and cancer therapy. These hydrogels hold significant potential for addressing critical challenges in these fields.

In the context of ischemic brain injury, a promising approach involves the combination of bone marrow mesenchymal stem cells (BMSCs) with rigid-flexible composite scaffolds. This synergistic approach has demonstrated improved therapeutic effects by reducing brain edema, infarct volume, and neurological deficits, while promoting neuronal proliferation and vascular growth. Such advances in the use of injectable hydrogels with BMSCs hold promise for the treatment of brain injuries [42].

Furthermore, injectable hydrogels have shown promise in tissue regeneration. For example, a sulfated cellulose nanocrystal (CNC-S) hydrogel loaded with vascular endothelial growth factor (VEGF) has been developed to facilitate tissue regeneration by promoting cellular infiltration and angiogenesis. This innovative hydrogel-based approach offers a potential solution for promoting tissue regeneration in various clinical scenarios [43].

The use of injectable hydrogels in the treatment of cardiovascular diseases has also been explored. A comprehensive review highlights the potential of injectable hydrogels as minimally invasive therapies for cardiac applications. The review discusses various strategies, including the combination of hydrogels with stem cells, cytokines, nanoparticles, exosomes, and genetic material. These strategies present exciting possibilities for improving cardiac function and promoting tissue regeneration in the context of cardiovascular diseases [44].

Moreover, injectable hydrogels demonstrate promise in targeted cancer therapy. A thermo-responsive nano-hydrogel loaded with triptolide has exhibited localized and sustained-release treatment of breast cancer. This approach displays enhanced cytotoxicity and anti-angiogenesis effects, underscoring the potential of injectable hydrogels in targeted cancer therapy. Such advancements hold considerable potential for improving cancer treatment outcomes [45].

Collectively, these studies highlight the remarkable capability of injectable hydrogels to address critical challenges in ischemic brain injury, tissue regeneration in cardiovascular diseases, and targeted cancer therapy. The development and utilization of injectable hydrogels in these areas pave the way for significant advancements in biomedical applications, improving patient outcomes.

### 7.2. Antibacterial

Injectable hydrogels have emerged as a versatile tool in various biomedical applications, including active cargo delivery, tissue regeneration, antibacterial applications, and bone reconstruction. One notable example of the potential of injectable hydrogels is found in the development of an antimicrobial colloidal hydrogel. This hydrogel incorporates graphene oxide (GO), thermo-sensitive nanogels (tNG), and silver nanoparticles (AgNPs). The resulting hydrogel possesses several desirable features, including tunable mechanical strength, responsive drug release, high antibacterial activity, temperature responsiveness, and self-healing properties. Such multifunctional characteristics make this hydrogel suitable for scaffold-based applications and antibacterial therapies, positioning it as a promising material with broad biomedical applications [151,152].

In the realm of bone regeneration and reconstruction, injectable bone substitutes composed of carrageenan (CG), nano-hydroxyapatite (nHA), and polymethylmethacrylate (PMMA) bone cement have demonstrated significant promise. These substitutes exhibit exceptional osteoblast adhesion, tissue regeneration potential, antimicrobial properties, remineralization capacity, as well as favorable physicochemical and mechanical performance. By offering a versatile solution for bone tissue engineering that does not solely rely on pharmaceutical drugs, these injectable bone substitutes represent a significant advancement in the field [153,154].

These examples highlight the potential of injectable hydrogels in advancing biomedical applications. Through their unique properties and versatile formulations, injectable hydrogels continue to pave the way for groundbreaking developments in the fields of active cargo delivery, tissue regeneration, antibacterial applications, and bone reconstruction.

### 7.3. Immiunotherapy

The field of injectable biomaterials has witnessed notable progress, resulting in innovative strategies for personalized cancer immunotherapy and tumor treatment. These advancements offer promising avenues to augment immune responses and effectively combat tumors.

One significant study involves the utilization of a self-assembled nano-vaccine platform that combines a conjugate of Toll-like receptor 7/8 agonist and tumor epitope (TLR7/8a-epitope) [46]. This nano-vaccine has demonstrated the capacity to enhance CD8 T-cell immunity, positioning it as a promising candidate for personalized immunotherapy in the treatment of melanoma tumors.

Another promising development revolves around the design of injectable smart hydrogels (ISHs) as a robust cancer vaccine platform. These hydrogels have the ability to recruit dendritic cells (DCs) and elicit tumor-specific immune responses, resulting in the eradication of melanoma tumors in preclinical mouse models. The ISHs offer a targeted and efficient approach to stimulate immune responses against cancer [47].

The integration of nanotechnology and biomaterials has played a pivotal role in advancing injectable biomaterials for cancer immunotherapy and tumor treatment [48]. Organic and polymeric carriers have also emerged as valuable tools in localized tumor chemo-immunotherapy, contributing to enhanced treatment effectiveness [49]. These technologies find diverse applications in diagnostics, drug delivery, cancer treatment, and tissue engineering, facilitating the development of personalized therapeutic strategies.

Furthermore, an injectable immunotherapy system based on a sodium alginate hydrogel has exhibited promise in inhibiting tumor recurrence and metastasis [50]. This system presents a potential strategy for adjuvant immunotherapy, providing supplementary support in cancer treatment.

Collectively, these advancements underscore the substantial potential of injectable biomaterials in personalized cancer immunotherapy and tumor treatment. The utilization of nanovaccine platforms, ISHs, organic and polymeric carriers, and sodium alginate hydrogels exemplifies the broad spectrum of applications and advantages offered by injectable biomaterials in the fight against cancer. These innovative approaches pave the way for more targeted, efficient, and personalized therapies in cancer immunotherapy [46,47,48,49,50].

### 7.4. Cartilage Repair

Significant strides have been made in recent investigations aimed at the development of injectable hydrogels for cartilage regeneration, presenting promising strategies to tackle the challenges associated with cartilage tissue engineering.

One notable approach involves the formulation of an injectable double-crosslinked hydrogel functionalized with kartogenin (KGN)-conjugated polyurethane nanoparticles (PN-KGN) and transforming growth factor beta3 (TGF-beta3) [51]. This hydrogel effectively promotes the migration and chondrogenesis of endogenous mesenchymal stem cells (MSCs), offering a viable in situ strategy for cartilage regeneration.

Another study focuses on an injectable biphasic semi-interpenetrating polymer network (SIPN) hydrogel impregnated with chondroitin sulfate (ChS) nanoparticles and ChS-loaded zein nanoparticles [52]. These nanoparticles are dispersed within injectable SIPNs developed by blending alginate with poly(vinyl alcohol) and calcium crosslinking. This hydrogel exhibits compatibility with chondrocytes and stimulates cartilage-specific gene expression and protein synthesis, demonstrating promise for the regeneration of hyaline cartilage.

Furthermore, a novel injectable biphasic hydrogel composed of partially hydrolyzed polyacrylamide (HPAM) crosslinked with chromium acetate and incorporating nanocrystalline hydroxyapatite (nHAp) has been developed [53]. This hydrogel supports cell viability and differentiation, positioning it as a prospective candidate for applications in cartilage tissue engineering.

In aggregate, these studies contribute to the advancement of injectable hydrogels for cartilage regeneration. By facilitating cell migration, chondrogenesis, and the expression of cartilage-specific genes, these hydrogels offer promising solutions for effective cartilage repair and regeneration.

### 7.5. Other Applications

The integration of nanomaterials with injectable self-healing hydrogels has yielded significant progress in therapies and regenerative medicine, showcasing their potential across a wide range of therapeutic domains.

In the field of cancer therapy, researchers have explored the combination of self-healing hydrogels with nanomaterials, such as Mn-Zn ferrite@mesoporous silica nanospheres and DOX [155]. This integration allows for tumor imaging and synergistic magnetothermal-chemo-chemodynamic therapy, offering efficient diagnosis and treatment for tumors.

Nano-engineered materials have been employed to enhance artificial extracellular matrices (ECMs) for improved cell scaffolds [156]. By modifying the mechanical properties of ECMs and providing dynamic stimuli, these materials enable wireless monitoring of cell status within cultures, leading to advancements in artificial ECMs.

For neurodegenerative diseases, a bioactive self-healing hydrogel based on a tannic-acid-modified gold nano-crosslinker has emerged as a potential injectable brain implant for treating Parkinson’s disease [157]. This hydrogel promotes neural stem cell proliferation and differentiation, possesses anti-inflammatory properties, and effectively restores motor function in a rat model of Parkinson’s disease.

Biodegradable polymers (BDPs) have gained prominence in various biomedical applications, including ophthalmic drug delivery [158]. Leveraging BDP-based implants, microneedles, and injectable particles enables targeted drug delivery to the ocular posterior segment, enhancing drug retention and bioavailability for ophthalmic treatments.

Advancements in nano- and micro-technologies have empowered researchers to exert precise control over hydrogel properties and functionalities for regenerative engineering [159]. These advancements hold significant implications for tissue engineering, encompassing musculoskeletal, nervous, and cardiac tissues.

Additionally, the development of an injectable conductive hydrogel shows promise in myocardial infarction (MI) treatment [160]. This hydrogel exhibits comparable myocardial conductivity and anti-fatigue performance. When loaded with plasmid DNA encoding endothelial nitric oxide synthase (eNOs) and adipose-derived stem cells (ADSCs), it improves heart function, thereby presenting a potential therapeutic strategy for MI treatment.

In the realm of dermal fillers, hyaluronic acid (HAc)-hydroxyapatite (HAp) composite hydrogels have been formulated as injectable fillers with enhanced biostability and bioactivity [161]. These composite fillers stimulate dermis recovery, collagen synthesis, and elastic fiber formation, positioning them as attractive candidates for long-lasting and multifunctional soft tissue augmentation.

Nanotechnology-based delivery vehicles are being investigated for the treatment of erectile dysfunction (ED) [162]. These vehicles exhibit promise in topical drug delivery, injectable gels, hydrogels for nerve regeneration, and encapsulation of drugs to enhance erectile function. Basic science studies underscore the potential of nanotechnology in developing therapies for ED, highlighting its utility in addressing male sexual dysfunction.

Lastly, a dual-network hydrogel based on an ionic nano-reservoir (INR) has been developed for sealing gastric perforations [163]. This hydrogel displays exceptional adhesion and mechanical properties, offering a potential solution for biomedical challenges such as gastric perforation treatment.

Collectively, these studies and advancements underscore the diverse applications and potential benefits arising from the integration of nanomaterials with injectable hydrogels in various therapeutic areas.

### 7.6. Perspective

The literature review highlights several significant trends and perspectives in the fields of drug delivery, tissue engineering, wound healing, cancer therapy, and therapeutic applications of injectable hydrogel systems. Injectable hydrogels have gained popularity as versatile platforms for drug delivery due to their gelation upon injection, which enables localized and sustained drug release [4,55,56,60,63,64,73,74,75,76,77,78,80,103]. Achieving targeted and localized drug delivery to specific sites, such as tumor cells or pathological areas, has been a major focus of research, and various strategies have been explored to achieve this objective [40,41,59,62,73,75,77].

Another essential aspect of drug delivery systems is sustained and controlled drug release, which can enhance therapeutic efficacy and minimize side effects. Various techniques have been employed to achieve sustained drug release, including hybrid formulations, nanogels, liposome-in-gel systems, and thermo-responsive hydrogels [3,40,59,62,63,72,73,75,80]. Furthermore, the integration of drug delivery systems with other therapeutic agents or techniques, known as combination therapies, has shown promise in achieving synergistic effects and improving treatment outcomes [40,56,59,60,74,75,77,80].

In the field of bone tissue regeneration and treatment of bone defects, hydrogel-based drug delivery systems have demonstrated significant potential. Various agents, such as peptides, calcium cations, micelles, and growth factors, have been incorporated into hydrogels to promote osteogenesis, bone formation, and bone regeneration [81,82,85,95,97,98]. Additionally, efforts have been focused on tailoring the mechanical properties and adaptability of hydrogels for specific applications, with the aim of enhancing mechanical strength, macroporosity, and adaptability to facilitate cell proliferation, implantation, and tissue engineering [94,95,97,100].

In the field of tissue engineering, the reviewed literature reveals patterns that highlight the versatility and potential of hydrogels. Aligned nanofiber yarns within hydrogel scaffolds have shown potential in promoting the alignment and elongation of nerve cells and other cell types, which is beneficial for nerve and bone tissue engineering [106,107]. Controlled release of bioactive substances from hydrogels has been demonstrated, enabling sustained release and promoting cell proliferation and tissue regeneration [6]. The incorporation of nanoparticles into hydrogels improves their mechanical and chemical properties, leading to enhanced cell viability, attachment, and proliferation, making them suitable for various tissue regeneration applications [108,112,116,125]. Injectable and thermosensitive hydrogels have been developed, offering advantages such as adjustable crosslinking, injectability, and support for cell proliferation and differentiation [8,115]. Interpenetrating network structures in hydrogels show promise for bone tissue engineering by providing porous structures, improved mechanical properties, and support for cell implantation [117,118,126].

In the field of bone tissue engineering and regeneration using hydrogel systems, controlled release systems have shown potential in enhancing osteogenic differentiation and bone formation by delivering bioactive factors over time [9,11]. Hydrogels have demonstrated biocompatibility and the ability to integrate with native tissues, making them suitable for tissue engineering and implantation [13]. Composite hydrogels with improved mechanical properties have been developed, exhibiting osteogenic potential and facilitating bone healing [19,25]. Additionally, hydrogels have shown promise in the treatment of various bone defects, including critical-size defects and infectious bone defects [10,23,132]. Advancements in rheological properties, injectability, and self-assembly capabilities have further improved the ease of application and in situ gel formation [22,26].

In the field of wound healing, the literature review reveals important trends and potential applications of injectable hydrogel systems. Thermo-sensitive hydrogels with antibacterial properties and tissue adhesion have shown potential for wound healing [134,139,146]. Hydrogels with controlled release capabilities and biocompatibility have demonstrated effectiveness in diabetic wound repair [135,138,142,147]. Various hydrogel formulations have been developed to promote wound healing, tissue regeneration, and control infections [28,136,140,141,147,148]. Antibacterial hydrogels have shown efficacy in inhibiting bacterial growth and managing drug-resistant infections [137,144,145,149]. Certain hydrogels also exhibit hemostatic abilities, contributing to efficient wound closure [143,147]. Injectable, self-healing, and versatile hydrogels have been designed for easy application and adaptation to different wound types [146,150].

In the field of cancer therapy, hydrogel systems have shown promise in improving therapeutic outcomes. The combination of multiple treatment modalities within hydrogels has been shown to have synergistic effects, leading to improved outcomes [30,32,35]. Hydrogels with controlled and temperature-dependent drug release capabilities have demonstrated targeted delivery and enhanced anti-tumor activity [34]. Additionally, hydrogels have shown potential in tumor ablation and inhibition through hyperthermia, singlet oxygen generation, and comprehensive treatment approaches [31,33,35]. The physicochemical properties and cytocompatibility of hydrogels further contribute to their clinical applicability in cancer therapy [30].

The integration of nanomaterials with injectable hydrogels opens up promising possibilities for therapeutic applications [49,156]. These hybrid systems have shown therapeutic effects in various diseases and conditions, including ischemic insult, CNS diseases, cancer, neurodegenerative diseases, ophthalmic treatments, tissue engineering, myocardial infarction, dermal fillers, erectile dysfunction, and gastric perforations. Nanomaterial-integrated hydrogels serve as versatile platforms for targeted drug delivery, tissue regeneration, immunotherapy, and implantable devices in biomedical contexts [49,156]. The combination of nanomaterials with injectable hydrogels enables synergistic treatment approaches, leading to improved outcomes in diagnosis, drug release, and therapeutic efficacy. The biocompatibility and biodegradability of these hydrogels make them suitable for long-term implantation or drug release without adverse effects [156,159]. Furthermore, injectable hydrogels integrated with nanomaterials hold promise for personalized therapies, tailoring treatments to individual patients and enhancing therapeutic effects against tumors [46,47].

It is also important to note that while the literature review provides valuable insights, further research and development are still necessary to overcome challenges and optimize the use of injectable hydrogel systems in clinical settings. Future studies could focus on refining the design and properties of hydrogels, exploring new combinations of therapeutic agents, advancing the understanding of their interactions with biological systems, and conducting rigorous preclinical and clinical trials to establish their safety and efficacy.

## 8. Conclusions

In conclusion, the development of nanostructured injectable hydrogel systems has advanced biomedical applications and expanded therapeutic possibilities. These materials play a crucial role in drug delivery, tissue engineering, wound management, cancer therapy, and bone regeneration. Injectable hydrogels provide precise control over drug release, targeted delivery, and improved mechanical properties. They show promise in various areas such as cardiac regeneration, joint diseases, ocular disorder treatment, and gynecological drug delivery. Furthermore, they have potential applications in diabetes treatment, wound healing, cancer therapy, and neuroregeneration. The integration of nanomaterials with injectable hydrogels has further broadened their use in advanced therapies and regenerative medicine. Ongoing advancements in hydrogel-based systems drive innovation in drug delivery and tissue engineering, leading to personalized treatments and improved patient outcomes. With their versatility, controlled release capabilities, and biocompatibility, injectable hydrogels offer a promising platform for addressing clinical challenges and revolutionizing medical treatments. They hold great potential for the future of regenerative medicine and contribute to the advancement of healthcare.

Disclosure: The authors partly used OpenAI’s large-scale language-generation model. The authors reviewed, revised, and edited the document for accuracy and take full responsibility for the content of this publication. The authors used Bing AI image creator to draw the graphical abstract.

Authors used Web of Science and PubMed to conduct literature search, used EndNote and over 20 essential keywords to screen the number of references down to 163.

## Figures and Tables

**Table 1:** Different hydrogel materials used for drug delivery.

Hydrogel Composition	Outcomes	Ref.
Polyurethane hydrogel and copper-substituted bioactive mesoporous glasses (Cu-MBGs)	Injectable hybrid formulations based on polyurethane hydrogel and Cu-MBGs enable simultaneous localized co-delivery of functional ions and drugs with sustained release profiles and tunable residence time at the pathological site.	[103]
Dexmedetomidine-loaded nano-hydrogel	Injectable nano-drug delivery system combined with Dexmedetomidine for thoracic paravertebral block significantly relieved pain, improved sleep quality, and reduced the need for remedial analgesia and side effects after thoracic surgery.	[3]
Nano-thermogel system of polyethylene glycol-polycaprolactone-polyethylene glycol (PEG-PCL-PEG) triblock with poly(lactic-co-glycolic acid) (PLGA) nanoparticles loaded p11 peptide	Controlled release of p11 peptide achieved with nano-thermogel system, showing potential for effective treatment of ocular disorders characterized by angiogenesis.	[4]
Hybrid silk hydrogel with carbon nanotubes	Hybrid silk hydrogel with carbon nanotubes enables localized, targeted, and on-demand delivery of anticancer drugs, reducing systemic side effects.	[40]
pH- and temperature-responsive hydrogels poly(ethylene glycol)-poly(beta-aminoester urethane)	Chondroitin sulfate nanogels incorporated into pH- and temperature-responsive hydrogels deliver cisplatin selectively to cancer cells, improving targeted therapy.	[41]
Urothelium-adherent, ion-triggered liposome-in-gel system	Liposome-in-gel system enhances drug penetration and adhesion in the bladder, showing prolonged drug retention and potential use in intravesical applications.	[58]
Composite liposome-in-gel system (gellan hydrogel)	Liposome-in-gel system delivers radiosensitizer paclitaxel to tumor site, enhancing the effect of concurrent radiotherapy and improving tumor volume reduction and animal survival.	[59]
Four-arm maleimide-functionalized polyethylene glycol (PEG-4MAL) hydrogel system	PEG-4MAL hydrogel acted as a mechanical pillow to protect the knee joint, inhibit cartilage degradation, and prevent osteophyte formation in an in vivo load-induced osteoarthritis mouse model.	[55]
Injectable hydrogel (amphiphilic polymers) system with tumor-targeting nano-micelles	The injectable hydrogel system sustainedly released tumor-targeting nano-micelles, which exhibited GSH-responsive drug release behavior, leading to enhanced antitumor efficiency and improved bioavailability of the drug.	[56]
Injectable thermosensitive photothermal-network hydrogel	The thermosensitive photothermal-network hydrogel demonstrated high photothermal conversion efficiency, reversible gel–sol transition, and on-demand drug release, enabling effective near-infrared-triggered photothermal-chemotherapy for breast cancer treatment.	[60]
Ultrasoft polymeric DNA networks of variable crystallinities	Ultrasoft self-supporting polymerized DNA networks with variable crystallinities showed tunable mechanical properties, pH-responsive drug release, and crystallinity-dependent antitumor efficacy, providing a favorable microenvironment for demand-localized drug delivery.	[62]
Sugar-based injectable thermoresponsive hydrogel	Injectable thermoresponsive hydroxypropyl guar-graft-poly(N-vinylcaprolactam) (HPG-g-PNVCL) hydrogel and its composite with nano-hydroxyapatite (n-HA) showed biocompatibility, thermoreversibility, slow drug release, and supported osteoblastic cell growth, making them potential scaffolds for bone tissue engineering.	[63]
Injectable polysaccharide hydrogel with hydroxyapatite and calcium carbonate	Injectable and degradable polysaccharide-based hydrogels integrated with hydroxyapatite and calcium carbonate show controlled gelation, enhanced mechanical properties, sustained drug release, antibacterial properties, and self-healing capabilities, making them promising for bone regeneration.	[64]
Injectable hydrogel nanomaterials (PNIPAAM with CS, APS and cross-linked with PEGDMA)	Continuous subcutaneous insulin infusion (CSII) showed better blood glucose control and lower incidence of hypoglycemia compared with multiple daily injections (MDI) in children with type 1 diabetes mellitus (T1DM).	[68]
Injectable liquid metal nanoflake hydrogel	The LM-doxorubicin nanoflake hydrogel with pH-triggered drug release shows enhanced therapeutic efficacy in preventing postoperative tumor relapse.	[72]
Bio-inspired fluorescent nano-injectable hydrogel prepared by copolymerization of N-isopropylacrylamide (NIPAM) and acrylic functionalized nucleobase (adenine)	The injectable hydrogel with a phase-separated structure enables sequential release of different drugs and exhibits fluorescence characteristics, making it suitable for dual drug delivery and imaging.	[73]
Injectable micromotor@hydrogel system	The micromotor@hydrogel drug delivery system protects micromotors and enables sustained release of erythromycin, exhibiting excellent antibacterial effect for the treatment of bacterial infections.	[74]
Nano polydopamine crosslinked thiol-functionalized hyaluronic acid hydrogel	The hydrogel, crosslinked using polydopamine nanoparticles, shows good injectability, mechanical stability, sustained drug release, and enhanced endothelial cell behavior, making it suitable for angiogenic drug delivery and tissue engineering.	[75]
poly(lactic-co-glycolic acid) (PLGA) MS loaded with melatonin(Mel) + Laponite hydrogels	The injectable micro-gel compound and nano-PM compound based on sustained-release microspheres provide stable and prolonged drug release, repair neural function, and reduce biomaterial loss for the treatment of spinal cord injury.	[76]
Injectable thermosensitive hydrogel (poly(d,l-lactide)-poly(ethylene glycol)-poly(d,l-lactide))containing erlotinib-loaded hollow mesoporous silica nanoparticles	The injectable ERT@HMSNs/gel composite provides sustained release of erlotinib, improves efficacy against NSCLC, and demonstrates an impressive balance between antitumor efficacy and systemic safety.	[77]
Injectable PEG-induced silk nanofiber hydrogel	The injectable silk fibroin nanofiber hydrogel, prepared using a dissolving technique and PEG, exhibits fast gelation, amorphous structure, and superior antibacterial properties, making it suitable for vancomycin delivery in tissue engineering.	[78]
Injectable hydrogel and nanoparticle system	The injectable hydrogel-nanoparticle system provides a promising approach for delivering microRNAs to cardiac tissue, improving cardiac function after myocardial infarction.	[80]
Sustained delivery system incorporating P24-loaded PLGA microspheres and nano-hydroxyapatite in composite hydrogel	The composite hydrogel with sustained P24 peptide release enhances bone tissue regeneration and shows potential for improving bone defect treatment in tissue engineering.	[81]
Injectable hydrogel (PLGA-PEG-PLGA) modified with hydroxyapatite particles	The hydrogel modified with nano- and core-shell hydroxyapatite particles enables controlled release of calcium cations, offering potential applications in bone regeneration.	[82]
Injectable thermo-sensitive hydrogel (hyaluronic acid-chitosan-g-poly(N-isopropylacrylamide)	The injectable hydrogel, combined with folic acid-conjugated graphene oxide (GOFA) nano-carrier, provides controlled and targeted intratumoral delivery of doxorubicin for breast cancer therapy.	[67]
Silica-triptorelin acetate depot	The silica-triptorelin acetate depot demonstrates sustained release of triptorelin, comparable to Pamorelin(R), and maintains equivalent pharmacodynamic effects with lower C_max_ values, offering potential for prolonged therapeutic effects.	[83]
Injectable 3-D nano-scaffold hydrogel	Mixing peptide-amphiphile (PA) with BMP-2 formed a transparent hydrogel that induced significant ectopic bone formation, offering potential for tissue regeneration.	[84]
Multi-functional calcitriol delivery system for osteoporotic bone regeneration based on poly(D, L-lactide)-poly(ethylene glycol)-poly(D, L-lactide) hydrogel	PDLLA-PEG-PDLLA hydrogel integrated with HA-D and PCL-PEG-NH2 micelles enabled sustained release of calcitriol, promoting osteogenesis and bone regeneration both in vitro and in vivo.	[85]
FRET-enabled monitoring of thermosensitive micellar hydrogel assembly (poly(epsilon-caprolactone-co-1,4,8-trioxa[4.6]spiro-9-undecanone)-b-poly(ethylene glycol)-b-poly(epsilon-caprolactone-co-1,4,8-trioxa[4.6]spiro-9-undecanone) triblock copolymer.	PECT triblock copolymer facilitated hydrogel formation and sustained release of micelles, allowing precise imaging of the fate of macro biodegradable materials and potential for co-delivery of therapeutic agents.	[86]
Thermosensitive micellar hydrogel (PECT triblock copolymer)	Injectable MHg depot composed of PECT micelles immobilized DOX and I-131-HA, enabling localized delivery, sustained release, and enhanced antitumor effect with reduced side effects.	[87]
Cell penetrable nano-polyplex hydrogel	Protamine-conjugated poly(organo-phosphazene) hydrogel forms after injection, releasing nano-polyplexes for effective siRNA delivery and long-term gene silencing on target site.	[88]
Biopolymer nano-network (Chitosan and dextran sulfate)	Colloidal nano-network made of chitosan and dextran sulfate encapsulates PA-13 antimicrobial peptide, protecting it from degradation, and delivers it locally, eliminating bacteria without impacting bioactivity.	[89]
Graphene oxide-containing self-assembling peptide hybrid hydrogels	GO-reinforced peptide hydrogels promote high cell viability and metabolic activity, showing potential as injectable scaffolds for in vivo delivery of nucleus pulposus cells.	[91]
Alginate nanohydrogels	BMP-2@ANH system promotes proliferation and differentiation of human bone marrow stromal cells into osteoblasts, offering a potential method for facilitating stem cell differentiation in vivo.	[92]
Nano-hybrid oligopeptide hydrogel	Topical delivery of docetaxel using DTX-CTs/Gel inhibited post-surgical tumor recurrence and enhanced cell death, showing promise for cancer therapy.	[104]
Chitosan-incorporated fatty acid vesicles hydrogel	Curcumin-loaded OCLAVs-CS hydrogel effectively reduced burst release, exhibited enhanced antioxidant activity, and can serve as an injectable or 3D printable drug delivery system.	[93]
Injectable two-step forming hydrogel (chitosan, collagen, hydroxypropyl-gamma-cyclodextrin and polyethylene glycol)	Hydrogel composed of chitosan, collagen, hydroxypropyl-gamma-cyclodextrin, and polyethylene glycol exhibited controlled release properties, adaptability for minimally invasive implantation, and support for cell proliferation.	[94]
Injectable poly(phosphazene) hydrogels with different anionic sidechains	Tunable hydrogel systems with optimized physical properties and BMP-2 release rates were identified, enabling effective bone regeneration in a critical-sized calvarial defect model.	[95]
Injectable hydrogel depot system using Exendin 4 (Ex-4) interactive and complex-forming polymeric ionic nanoparticles	The hydrogel system demonstrated prolonged release of Exendin 4 (Ex-4), offering potential as a long-term effective and reproducible treatment for type 2 diabetes mellitus.	[96]
Two-in-one injectable micelleplex-loaded thermogel system composed with polymerization of poly(ethylene glycol), poly(propylene glycol), and poly(3-hydroxybutyrate)	The novel nanoparticle-hydrogel system enabled prolonged release of pDNA micelleplexes, indicating its potential for sustained gene delivery applications.	[105]
Injectable alginate-based hydrogel cross-linked via the regulated release of divalent ions from the hydrolysis of D-glucono-delta-lactone	The hydrogel exhibited improved mechanical properties through the slow release of divalent ions from D-glucono-delta-lactone, making it suitable for bone tissue engineering applications.	[97]
Injectable bone regeneration composite (IBRC) with nano-hydroxyapatite/collagen particles in an alginate hydrogel carrier	The controlled release of rhBMP-2 from IBRC promoted bone formation, highlighting its potential as a bone defect repair material for clinical applications.	[98]
Moldable/injectable calcium phosphate cement (CPC) composite scaffolds	Strong, macroporous CPC scaffolds were developed, suitable for bone regeneration, cell delivery, and growth factor release, with potential applications in dental, craniofacial, and orthopedic reconstructions.	[100]
Injectable and quadruple-functional hydrogel (folate/polyethylenimine-conjugated poly(organophosphazene) polymer) encapsulated with siRNA and Au-Fe_3_O_4_ nanoparticles	The hydrogel-based delivery method improved tumor targeting efficiency compared with intravenous delivery, enabling sustained release, passive targeting, active targeting, and magnetic targeting for enhanced therapeutic effects.	[101]
Injectable thermosensitive polymeric hydrogel of poly(organophosphazene) combined with superparamagnetic iron oxide nanoparticles	The designed injectable hydrogel allowed controlled release of TRAIL/SPION nanocomplex under hyperthermia, resulting in enhanced cytotoxicity against TRAIL-resistant cancer cells and significant tumor reduction in vivo.	[102]

Abbreviations: PNIPAAM, Poly(N-isopropylacrylamide); PEGDMA, Polyethylene glycol dimethacrylate; nHA, nano-hydroxyapatite; PLGA, poly(lactide-co-glycolide); PEG, poly(ethylene glycol); FRET, Fluorescence resonance energy transfer; PECT, poly(epsilon-caprolactone-co-1,4,8-trioxa[4.6]spiro-9-undecanone)-poly(ethyleneglycol)-poly(epsilon-caprolactone-co-1,4,8-trioxa[4.6]spiro-9-undecanone).

**Table 2:** Different hydrogel materials used for tissue engineering.

Hydrogel Composition	Outcomes	Ref.
Core-shell scaffold based on aligned conductive nanofiber yarns (NFYs) within a methacrylated gelatin (GelMA) hydrogel	Aligned nanofiber yarns within a hydrogel scaffold induce neurite alignment and extension, promoting the alignment and elongation of nerve cells, offering potential for nerve tissue engineering applications.	[106]
In situ forming thermosensitive chitosan-glycerol phosphate hydrogel loaded with risedronate and nano-hydroxyapatite	The prepared hydrogel formulation with risedronate and nano-hydroxyapatite showed sustained drug release, enhanced Saos-2 cell proliferation, alkaline phosphatase activity, and calcium deposition, making it a promising option for bone tissue engineering.	[6]
Protein-based hydrogels derived from natural tissues	Investigating the nano-/micro-structure and composition of protein-based hydrogels derived from natural tissues is crucial for their widespread use in tissue engineering and regenerative medicine.	[129]
Calcium alginate-gum tragacanth hydrogels incorporated with cobalt-doped nano-hydroxyapatite	The hydrogels exhibited enhanced swelling, degradation, diffusion, long-term viability of encapsulated cells, osteogenic differentiation, and angiogenic properties, making them suitable for bone tissue engineering applications.	[107]
Chemically crosslinked collagen/chitosan/hyaluronic acid hydrogels	Optimization of the hydrogel composition showed that using high concentrations of crosslinking agent and adjusting the hyaluronic acid content resulted in hydrogels with compact structure, good mechanical properties, prolonged degradation profile, and suitable biocompatibility for bone regeneration applications.	[7]
Injectable PCL-PEG-PCL-Col/nHA hydrogels	PCL-PEG-PCL-Col/nHA hydrogels showed successful integration of collagen and nano-hydroxyapatite, delayed biodegradation rate, no prominent pro-inflammatory response, and increased expression of CD31 and IL-10, indicating biocompatibility for hard tissue regeneration.	[108]
Enzymatically crosslinked CMC/gelatin/nHAp injectable gels	The enzymatically crosslinked injectable gels exhibited rigidity, adjustable crosslinking degree and strength, increased pore sizes with higher gelatin concentration, and support for osteoblast cell proliferation and differentiation, making them suitable for in situ bone tissue engineering applications.	[8]
Injectable semi-interpenetrating network hydrogel with chondroitin sulfate nanoparticles (ChS-NP)s and nanohydroxyapatite (nHA)	The gradient hydrogel construct demonstrated mineralized subchondral and chondral zones, higher osteoblast proliferation in the subchondral zone, porous structure with gradient interface, layer-specific retention of cells, and in vivo osteochondral regeneration with hyaline cartilage formation and subchondral bone integration.	[109]
Alginate dialdehyde-gelatin scaffolds with zirconium oxide nanoparticles	Incorporation of ZrO_2_ nanoparticles into alginate-gelatin hydrogels enhances mechanical and chemical properties. Nanocomposite hydrogels exhibit improved swelling behavior, controlled biodegradation, cell viability, and attachment, making them suitable for cartilage tissue regeneration.	[112]
Alginate-O-carboxymethyl chitosan/nano fibrin composite hydrogels	Alginate/O-CMC hydrogel blend demonstrated superior properties for tissue engineering applications, supporting the survival, adhesion, proliferation, and differentiation of adipose-derived stem cells.	[113]
Injectable carrageenan nanocomposite hydrogel	Carrageenan nanocomposite hydrogel incorporated with whitlockite nanoparticles and an angiogenic drug promoted osteogenesis and angiogenesis in vitro, showing potential for bone tissue engineering.	[114]
Injectable thermosensitive hydrogel made of poly(ethylene glycol)-poly(epsilon-caprolactone)-poly(ethylene glycol) (PECE) and nanohydroxyapatite (n-HA)	Thermosensitive hydrogel nanocomposites exhibited good thermosensitivity, injectability, and 3D network structure, making them promising for injectable orthopedic tissue engineering.	[115]
Chitosan/collagen hydrogels nano-engineered with functionalized single-wall carbon nanotubes	Integration of COOH-SWCNTs into chitosan and collagen hydrogels increased mechanical strength, bioactivity, and potential for bone tissue engineering and regenerative medicine.	[116]
Nano-hydroxyapatite/glycol chitosan/hyaluronic acid composite hydrogel	Composite hydrogel exhibited porous structure, enzymatic degradation, and cytocompatibility, making it suitable for bone tissue engineering applications.	[117]
Laponite nanoparticle-associated silated hydroxypropylmethyl cellulose hydrogel	Incorporation of laponites into silated hydroxypropylmethyl cellulose hydrogel resulted in an interpenetrating network that improved mechanical properties without compromising cytocompatibility, oxygen diffusion, or chondrogenic cell functionality.	[118]
Nano SIM@ZIF-8-modified injectable high-intensity biohydrogel composed of composed of poly (ethylene glycol) diacrylate (PEGDA) and sodium alginate (SA) + nano simvastatin-laden zeolitic imidazolate framework-8	nSZPS hydrogel stimulates osteogenic differentiation, inhibits adipogenic differentiation, exhibits excellent injectability, mechanical strength, and promotes bone regeneration in hyperlipidemic microenvironments.	[119]
Nano-silicate-reinforced and SDF-1alpha-loaded gelatin-methacryloyl hydrogel	GelMA-SN-SDF-1alpha hydrogel demonstrates injectability, controlled release of SDF-1alpha, MSC migration and homing, and excellent bone regeneration ability in critical-sized calvaria defects.	[120]
Succinylated gelatin cross-linked with aldehyde heparin formed nanoparticles, which were mineralized with hydroxyapatite (mineralized heparin-gelatin nanoparticles)	These nanoparticles may enhance the mechanical properties of injectable hydrogels for bone regeneration.	[122]
Injectable platelet-rich plasma (PRP)/cell-laden microcarrier/hydrogel composite system	Gelatin methacryloyl (GelMA) and chitosan hydrogels were used to prepare scalable interpenetrating network GelMA/chitosan-microcarriers (IGMs) loaded with PRP and dermal papilla cells (DPCs). The composite system promoted DPC viability, hair inducibility, and hair follicle regeneration.	[123]
Polysaccharide-based injectable hydrogel compositing nano-hydroxyapatite	N-carboxyethyl chitosan (NCEC) and oxidized dextran (ODex) were cross-linked via Schiff base linkage to form an injectable hydrogel. The hydrogel, composited with nano-hydroxyapatite (nHAP), exhibited interconnected porous structure and showed excellent bone repair effect in vivo.	[124]
Bioactive glass nanoparticle-incorporated triblock copolymeric injectable hydrogel	Injectable hydrogel with bioactive glass nanoparticles showed good gelling and injectability properties, excellent swelling properties, enhanced bone cell proliferation, ALP activity, and apatite mineralization for accelerated in vitro osteogenesis.	[125]
Nano-fibrillar hybrid injectable hydrogel with heterotypic collagen fibrils	Injectable hydrogel with semi-interpenetrating networks of heterotypic collagen fibrils in a glycol-chitosan matrix showed nano-fibrillar porous structure, mechanical stability, prolonged half-life, and support for cell implantation.	[126]
Visible-light-mediated nano-biomineralization of customizable tough hydrogels	Rapid preparation of biomineralized tough hydrogels with improved mechanical and biological properties under visible light irradiation, suitable for customizable skin repair and bone regeneration.	[128]

Abbreviations: PCL-PEG-PCL-Col/nHA, Poly(ε-caprolactone)-poly(ethylene glycol)-poly(ε-caprolactone)/collagen/nano-hydroxyapatite; CMC, carboxymethyl-chitosan; SDF-1alpha stromal cell-derived factor-1 alpha.

**Table 3:** Different hydrogel materials used for bone repair and osteogenesis.

Hydrogel Composition	Outcomes	Ref.
RADA16 peptide hydrogel filled with porous calcium sulfate/nano-hydroxyapatite (CaSO_4_/HA) composite biomaterial	Controlled and sustainable release of bFGF for more than 32 days from RADA16/CaSO_4_/HA composite biomaterial, leading to enhanced osteogenic differentiation in vitro and improved bone formation in vivo.	[9]
Injectable chitin-PLGA hydrogel containing bioglass nanoparticles (nBG) or whitlockite nanoparticles (nWH) with FGF-18	CGnWHF (nWH + FGF-18 containing CG) showed the highest osteogenic potential and near-complete bone regeneration in critical-sized defect region compared to other groups, indicating its potential for craniofacial bone defects.	[10]
GelMA-HAMA/nHAP composite hydrogel with human-urine-derived stem cell exosomes	Composite hydrogel with controlled release of USCEXOs promotes osteogenesis and angiogenesis, enhancing bone regeneration in vivo.	[11]
Injectable bone regeneration composite (IBRC) with nano-hydroxyapatite/collagen (nHAC) particles in alginate hydrogel carrier	IBRC exhibited controllable degradability and biocompatibility, making it a promising material for bone repair and tissue engineering.	[12]
poly (caprolactone)-poly(ethylene glycol)-poly(caprolactone) + gelatin and nano-hydroxyapatite	Hydrogels showed successful integration of Gel and nHA, lacked inflammation, and exhibited biocompatibility without toxic effects in in vivo conditions.	[13]
nano-hydroxyapatite hybrid methylcellulose hydrogel carrying bone mesenchymal stem cells	Addition of nHA to MC hydrogel enhances cell survival, osteogenic differentiation, and remediation efficiency in vivo.	[14]
Thermo-sensitive PEG-PCL-PEG copolymer/collagen/n-HA hydrogel composite	Composite hydrogel exhibits thermosensitivity, biocompatibility, and better performance in guided bone regeneration compared to self-healing processes.	[15]
Injectable polysaccharide hydrogel-loaded nano-hydroxyapatite	Hydrogel/hydroxyapatite composite scaffold enhances new bone area and alveolar ridge promotion, while promoting soft tissue healing.	[16]
TME-modulated hydrogel (MBGN/Gel/OCS)	Hydrogel interferes with tumor microenvironment, overcomes cancer resistance, and promotes sustained drug release and osteogenesis.	[17]
Injectable hydrogel containing cisplatin (DDP) and polydopamine-decorated nano-hydroxyapatite (DDP/PDA/nHA)	Exhibits dual functions of tumor therapy and bone regeneration, effectively ablating tumor cells and inducing bone regeneration.	[18]
Light-cured hyaluronic acid composite hydrogels (nano-HA/chitosan)	Enhance mechanical properties and osteogenic potential, promising for bone regeneration applications.	[19]
Nanocellulose reinforced alginate hydrogel(AC) that carried beta-tricalcium phosphate (beta-TCP) nano-powder and liver-derived extracellular matrix (ECM) from porcine	ETAC Show enhanced cytocompatibility, accelerated bone regeneration, and improved healing quality compared to TAC and AC beads.	[20]
Chitin-CaSO_4_-nFibrin gel	Demonstrates improved rheology, angiogenic potential, and osteo-regeneration compared to chitin control.	[21]
Silk-hydroxyapatite composite	Exhibits injectability, thixotropy, and osteodifferentiation potential, supporting improved osteogenesis and bone defect healing.	[22]
nHA@Gel/ADA hydrogel with gelatin, alginate dialdehyde, Ca^2+^, borax, and nano-sized hydroxyapatite	Promotes efficient repair of critical-size skull bone defects and supports macrophage-BMSC crosstalk.	[23]
Composite hydrogel system incorporating PLGA-BMP-2 and PLA-17 beta-estradiol microspheres in a hydrogel core	Shows controlled release, refilling of bone defects, and regeneration in osteoporotic rats.	[24]
Injectable hydrogel with chitosan/beta-glycerophosphate disodium salt (CS/GP) and chitin nano-whiskers (CNWs)	Exhibits improved mechanical properties, gelation speed, and biocompatibility, suitable for tissue engineering scaffold applications.	[25]
PDH/mICPN hydrogel composed of DMAEMA, HEMA, CaP nanoparticles (ICPNs), and poly-L-glutamic acid (PGA)	Self-assembles in situ, demonstrating enhanced mechanical strength, cell adhesion, and osteodifferentiation for bone regeneration.	[26]
Injectable bone regeneration composite (IBRC) with calcium alginate hydrogel matrix carrying nano-hydroxyapatite/collagen	Demonstrates structural homogeneity, good biocompatibility, and the ability to promote bone healing.	[27]
Biomimetic/osteoinductive injectable hyaluronan-based hydrogel loaded with nano-hydroxyapatite crystals (Hya/HA)	Shows potential for enhancing bone architecture, with an osteoinductive effect and improved bone density and architecture in the rabbit distal femur.	[130]
Demineralized dentin matrix hydrogel (DDMH)	Exhibits a porous structure and supports viability and differentiation of BMMSCs, with potential for promoting bone formation. A 50% concentration of DDMH shows promising results.	[131]
Gellan gum (GG)-based injectable hydrogel loaded with chlorhexidine (CHX) and nanohydroxyapatite (nHA)	Demonstrates superior biocompatibility, mechanical strength, osteogenic properties, and antibacterial effect against *E. faecalis*. Shows potential for treating infectious bone defects.	[132]

Abbreviations: GelMA-HAMA/nHAP, Gelatin-methacrylate-Hyaluronic acid methacrylate/nano-hydroxyapatite; PEG-PCL-PEG, poly(ethylene glycol)-poly(ε-caprolactone)-poly(ethylene glycol); TME, Tumor microenvironment; MBGN/Gel/OCS, modulated hydrogel composed of bio-responsive drug-loaded mesoporous bioactive glass nanoparticles/gelatin/oxidized chondroitin sulfate; DMAEMA, dimethylaminoethyl methacrylate; HEMA, 2-hydroxyethyl methacrylate.

**Table 4:** Different hydrogel materials used for wound healing.

Hydrogel Composition	Outcomes	Ref.
PLEL-nBG-QCS-C hydrogel: poly(d,llactide)-poly(ethylene glycol)-poly(d,l-lactide) PLEL, nano-scaled bioactive glass (nBG), and catechol modified quaternized chitosan (QCS-C)	Exhibits thermo-sensitivity, antibacterial properties, tissue adhesion, and accelerates wound healing	[134]
Chitosan-CMC-g-PF127 injectable hydrogels loaded with nano-curcumin	Show controlled release, biocompatibility, and promote diabetic wound repair	[135]
BT-CTS thermogel: Injectable thermosensitive hydrogel with black titania nanoparticles (B-TiO_2_-x) in chitosan matrix	Provides effective tumor therapy, wound closure, and tissue regeneration for skin tumors	[136]
Injectable silver-gelatin-cellulose ternary hydrogel dressing with aminated silver nanoparticles	Exhibits antibacterial properties and enhances cutaneous wound healing in infant nursing care	[137]
ZnO-Ber/H: Berberine-modified ZnO nano-colloids hydrogel	Promotes diabetic wound healing by enhancing wound healing rate, regulating antioxidant stress factors, downregulating inflammatory factors, and promoting the expression of vascular and epithelial tissue-related factors	[138]
CG/PDA@Ag hydrogel: Cationic guar gum hydrogel encapsulating Polydopamine NPs with Ag (PDA@Ag)	Combines high photothermal conversion efficiency and inherent antibacterial ability, demonstrating superior antibacterial efficacy for photothermal antibacterial therapy	[139]
Injectable alginate nanocomposite hydrogel containing nano-sized calcium fluoride particles	Enhances bioactivity, antibacterial property, cell proliferation, migration, and extracellular matrix deposition for accelerated wound healing	[28]
GG-MA/Laponite hydrogel: Gellan gum methacrylate (GG-MA) combined with laponite (R) XLG	Shows improved mechanical properties and potential as wound dressing materials for infected wounds	[29]
Nano-curcumin/CCS-OA hydrogel: In situ injectable hydrogel composed of curcumin, N,O-carboxymethyl chitosan, and oxidized alginate	Accelerates wound healing by promoting re-epithelialization and collagen deposition in rat dorsal wounds	[140]
KA hydrogel: Injectable oxidized alginate/carboxymethyl chitosan hydrogel functionalized with keratin nanoparticles (Ker NPs) and nanosized-EGCG covered with Ag nanoparticles (AE NPs)	Accelerates wound healing, particularly in the early stage, and improves the thickness of renascent epidermis	[141]
M@M-Ag-Sil-MA hydrogel: Photocurable methacryloxylated silk fibroin hydrogel (Sil-MA) co-encapsulated with metformin-loaded mesoporous silica microspheres (MET@MSNs) and silver nanoparticles (Ag NPs)	Resolves immune contradiction in diabetic wounds, promotes fibroblast migration and endothelial cell angiogenesis, and accelerates diabetic wound healing in a diabetic mouse model	[142]
RAAS hydrogel: Injectable hydrogel adhesive with rapid adhesion to wet tissues and anti-swelling properties.	Achieves rapid adhesion to wet tissues, exhibits excellent anti-swelling properties, and demonstrates fast hemostasis and stable adhesion strength in diverse hemorrhage models	[143]
GA@AgNPs-SA hydrogel: Injectable sodium alginate hydrogel loaded with gallic acid-functionalized silver nanoparticles (GA@AgNPs)	Exhibits long-term antimicrobial effect, reduces inflammatory response, and accelerates the repair of bacteria-infected wounds through sustained release of silver ions and promotion of angiogenesis	[144]
Injectable hydrogel with Ag-doped Mo2C-derived polyoxometalate (AgPOM) nanoparticles, urea, gelatin, and tea polyphenols (TPs)	Exhibits antibacterial activity, accelerates wound healing, and shows potential as a therapeutic agent for drug-resistant bacteria-infected wounds	[145]
CMCS-brZnO hydrogel: Injectable hydrogel synthesized by incorporating fusiform-like zinc oxide nanorods (brZnO) into carboxymethyl chitosan (CMCS)	Demonstrates injectability, self-healing, tissue adhesion, antibacterial activity, and promotion of wound healing through sustained release of antibacterial Zn(^2+^) ions	[146]
Silk fibroin-hyaluronic acid based injectable hydrogel incorporated with mace-like Au-CuS heterostructural nanoparticles (gAu-CuS HSs)	Enhances hemostasis, exhibits antibacterial activity, regulates cytokine expression, promotes angiogenesis, and accelerates wound healing, making it a promising strategy for diabetic wound healing	[147]
PH/sFDM hydrogel containing nano-sized suspended formulation and Pluronic F127/hyaluronic acid (HA)	Promotes neovessel formation, collagen deposition, blood reperfusion, and reduces necrosis and fibrosis in cutaneous wound and hindlimb ischemia models	[148]
Self-assembling hydrogels based on thiolated hyaluronic acid (HA-SH) and bioactive silver-lignin nanoparticles (Ag@Lig NPs)	Inhibits proteolytic enzymes, oxidative enzymes, and bacteria, while promoting tissue remodeling and skin integrity restoration in chronic wounds	[149]
Guar gum-grafted-polyacrylamidoglycolic acid (GG-g-PAGA) polymer-based silver nanocomposite (AgNC) hydrogels	Exhibits self-healing ability, injectability, stretchability, flowability, high swelling, porosity, mechanical behavior, and biodegradability, suitable for wound-healing applications	[150]

Abbreviations: sFDM, Human fibroblast derived matrix in nano-sized suspended formulation.

**Table 5:** Different hydrogel materials used for photothermal therapy.

Hydrogel Materials and Composition	Outcomes	Ref.
Chitosan-based injectable in-situ-forming hydrogels containing dopamine-reduced graphene oxide (DOPA-rGO) and resveratrol (RES)	Exhibits injectability, in situ gelation, suitable physicochemical properties, and good cytocompatibility, and significantly enhances the efficacy of chemo-photothermal therapy in breast cancer cells.	[30]
In situ injectable PEG hydrogel system formulated with albumin nanoparticles	Exhibits hyperthermia, singlet oxygen ((1)O(2)) generation, and enhanced killing of tumor cells, showing potential for ablation of poorly responsive hypoxic tumors.	[31]
Injectable and biodegradable nano-photothermal DNA hydrogel	Exhibits improved penetration, sensitivity to photothermal therapy (PTT) and photodynamic treatment (PDT), easy cellular uptake, enhanced anti-tumor activity, and reduced drug resistance, providing a safe and efficient supplement for cancer therapy.	[32]
Injectable and self-healing nanocomposite hydrogel loaded with needle-like nano-hydroxyapatite (HAP) and graphene oxide (GO)	Effectively inhibits tumor cell proliferation, realizes the synergistic effect of photothermal therapy, and shows potential as an effective treatment approach for tumors.	[33]
Injectable in situ intelligent thermo-responsive hydrogel with glycyrrhetinic acid (GA)-conjugated nano graphene oxide (NGO)	Exhibits sustained and temperature-dependent drug release, enhanced anti-tumor activity when combined with laser irradiation, and shows potential for clinical treatment of malignant tumors.	[34]
Injectable nano-composite hydrogel based on hyaluronic acid-chitosan derivatives	Demonstrates tumor inhibition through a comprehensive approach of photothermal therapy, chemotherapy, and anti-inflammatory effects.	[35]
Silk fibroin nanofiber (SF) hydrogel system complexed with upconversion nanoparticles and nano-graphene oxide (SF/UCNP@NGO)	Shows potential for tumor imaging and therapy, with excellent biocompatibility, efficient cancer cell ablation, and outstanding antitumor efficacy.	[36]
Injectable, near-infrared (NIR)/pH-responsive nanocomposite hydrogel	Demonstrates potential as a long-term drug delivery platform for chemophotothermal synergistic cancer therapy, reducing adverse effects and enabling prolonged drug retention in the tumor region.	[37]
Thermosensitive TMPO-oxidized lignocellulose/cationic agarose hydrogel	Shows potential for photothermal therapy in melanoma, with short gelation time, high mechanical strength, efficient drug release, and reduced cytotoxicity with laser light irradiation.	[39]

## Data Availability

Not applicable.
